# Meso scale discovery-based assays for the detection of aggregated huntingtin

**DOI:** 10.1371/journal.pone.0213521

**Published:** 2019-03-26

**Authors:** Wolfgang Reindl, Barbara Baldo, Jana Schulz, Isabell Janack, Ilka Lindner, Markus Kleinschmidt, Yalda Sedaghat, Christina Thiede, Karsten Tillack, Christina Schmidt, Isabell Cardaun, Tom Schwagarus, Frank Herrmann, Madlen Hotze, Georgina F. Osborne, Simone Herrmann, Andreas Weiss, Celina Zerbinatti, Gillian P. Bates, Jonathan Bard, Ignacio Munoz-Sanjuan, Douglas Macdonald

**Affiliations:** 1 Evotec AG, Hamburg, Germany; 2 Dept. Neurodegenerative Disease, Huntington’s Disease Centre and Dementia Research Institute, Institute of Neurology, University College London, London, United Kingdom; 3 CHDI Management/CHDI Foundation, Los Angeles, CA, United States of America; Emory University, UNITED STATES

## Abstract

Huntington’s disease (HD) is a monogenic neurodegenerative disorder caused by an expansion of the CAG trinucleotide repeat domain in the huntingtin (*HTT*) gene, leading to an expanded poly-glutamine (polyQ) stretch in the HTT protein. This mutant HTT (mHTT) protein is highly prone to intracellular aggregation, causing significant damage and cellular loss in the striatal, cortical, and other regions of the brain. Therefore, modulation of mHTT levels in these brain regions in order to reduce intracellular mHTT and aggregate levels represents a direct approach in the development of HD therapeutics. To this end, assays that can be used to detect changes in HTT levels in biological samples are invaluable tools to assess target engagement and guide dose selection in clinical trials. The Meso Scale Discovery (MSD) ELISA-based assay platform is a robust and sensitive method previously employed for the quantification of HTT. However, the currently available MSD assays for HTT are primarily detecting the monomeric soluble form of the protein, but not aggregated species. In this study, we describe the development of novel MSD assays preferentially detecting mHTT in an aggregated form. Recombinant monomeric HTT(1–97)-Q46, which forms aggregates in a time-dependent manner, was used to characterize the ability of each established assay to distinguish between HTT monomers and HTT in a higher assembly state. Further validation of these assays was performed using brain lysates from R6/2, zQ175 knock-in, and BACHD mouse models, to replicate a previously well-characterized age-dependent increase in brain aggregate signals, as well as a significant reduction of aggregate levels in the striatum following mHTT knockdown with a CAG-directed allele-specific zinc-finger repressor protein (ZFP). Lastly, size exclusion chromatography was used to separate and characterize HTT species from brain tissue lysates to demonstrate specificity of the assays for the fractions containing aggregated HTT. In summary, we demonstrate that the newly developed assays preferentially detect aggregated HTT with improved performance in comparison to previous assay technologies. These assays complement the existing MSD platform assays specific for soluble HTT monomers, allowing for a more comprehensive analysis of disease-relevant HTT species in preclinical models of HD.

## Introduction

Huntington’s disease (HD) is a neurodegenerative genetic disorder that leads to motor dysfunction and cognitive decline [1]. The neuropathology is most prominently characterized by neuronal loss and atrophy of the caudate nucleus and putamen regions of the striatum [2, 3]. HD is caused by the abnormal expansion of CAG trinucleotide repeats within exon 1 of the huntingtin (*HTT*) gene, which leads to the expansion of the poly-glutamine (polyQ) stretch located in the N-terminus of the HTT protein [4].

The onset and severity of the disease correlates with the repeat length of the polyQ stretch: individuals with 35 and less CAGs do not show any symptoms, while manifestation of the disease starts from 36 CAGs onward [5]. The expanded polyQ stretch increases the propensity of the HTT protein to form aggregates that accumulate within both the nucleus and cytoplasm of cells, most prominently in neurons. The rate of mHTT aggregation is proportional to the increase in CAG repeat length and age [6], and correlates with susceptibility to cell death and loss of neuronal tissue [7, 8]. It has been previously shown that shutting down mHTT expression in a conditional HD mouse model leads to the reduction of nuclear and extranuclear HTT aggregates, and to a concomitant improvement of cognitive deficits [9]. Therefore, modulation of mHTT and mHTT aggregate levels represents a promising therapeutic approach for HD patients. For this reason, multiple HTT-lowering agents such as antisense oligonucleotides (ASO), small interfering RNAs (siRNA), micro RNAs (miRNA), and zinc finger repression proteins (ZFP) are in or under development for clinical testing based on promising results generated in preclinical models.

ASOs that catalyze RNase H-mediated degradation of HTT mRNA were reported to produce sustainable suppression of mHTT expression and prevent brain mass loss in HD rodent models [10]. In addition, single-stranded siRNAs (ss-siRNAs) were also shown to efficiently inhibit mHTT expression throughout the brain of heterozygous *Hdh*Q150 mice [11]. Adeno-associated virus (AAV)-mediated delivery of miRNAs has been reported to lower mHTT expression, reduce aggregate levels and reverse disease symptoms in YAC128 mice [12]. AAV-mediated delivery of shRNA against HTT also leads to marked reduction in HTT aggregate levels in the transduced striatum of zQ175 mice [13]. A relatively new field is the application of ZFPs that selectively target mutant HTT. In this context, AAV-mediated ZFP expression reduces HTT aggregates and improves behavioral deficits in R6/2 and zQ175 mice [14, 15].

As these HTT-lowering therapies advance to clinical testing, assessing their value for HD patients requires reliable assays that can be used to measure HTT levels in relevant biological samples for assessment of target engagement and efficacy. Several assay systems using time-resolved fluorescence resonance energy transfer (TR-FRET), ELISAs, ELISA-based Meso Scale Discovery (MSD) electrochemiluminescence assays, or the Erenna Singulex platforms have been developed for the quantification of soluble monomeric and mutant HTT protein species [16–20]. Nonetheless, only a limited number of assays for selective and quantifiable detection of oligomeric or aggregated HTT have been developed to date. For example, AGERA (agarose gel electrophoresis for resolving aggregates) is a qualitative approach to assess HTT aggregates in biological samples [21]. A Seprion ligand ELISA assay, which utilizes a proprietary aggregate-capturing reagent, has been developed for detection of HTT aggregates [22]. In addition, two homogeneous TR-FRET-based immunoassays were established for selective quantification of HTT aggregates in cell and tissue homogenates [23].

The MSD assay platform has emerged as a versatile, sensitive, and robust technique for detection of HTT. Multiple combinations of capturing and detection antibodies allow for the quantification of different forms of HTT [19]. Here, we describe the development of novel MSD assays preferentially detecting aggregated HTT that complement the existing assays for the quantification of soluble HTT monomers. These assays represent new platform tools for the analysis of preclinical HD models. They could be used for measuring prevention of aggregate formation and reversal of formed aggregates, or evaluation of HTT protein life cycle. As an immuno-based assay system, the MSD technology requires the availability of selective antibodies. MW8, a mouse monoclonal antibody generated against soluble and aggregated human exon 1-Q67 as immunogen, has been previously applied for staining of HTT aggregates by immunohistochemistry [24, 25] and aggregate detection by TR-FRET assays [23]. Using MW8 in various combinations with other anti-HTT antibodies, we were able to develop HTT aggregation MSD assays that showed equal or improved performance in comparison to existing assay technologies. These assays were able to detect a well-characterized age-dependent increase in HTT aggregate signal in the brain of HD mouse models [13, 22, 26], and a significant reduction in HTT aggregates after mHTT knockdown with ZFP in the zQ175 mouse striatum [15]. Additionally, analysis of HD mouse brain homogenates after separation of HTT monomers and aggregates by size exclusion chromatography confirmed the preferential aggregate binding of the newly-developed assays. In summary, the novel aggregate-detecting MSD assays developed here can be used to expand the available techniques for HTT detection and allow for a more comprehensive analysis of HTT protein species in biological samples.

## Materials and methods

### Anti-HTT antibodies

Generation of the following antibodies was previously described: rabbit polyclonal antibody CHDI-90000147 (pAb147) binding to amino acids 37–53 within the poly-proline domain of mouse HTT [19]; mouse monoclonal antibody CHDI-90000895 (MW1) binding to the polyQ stretch of HTT [24]; mouse monoclonal antibody CHDI-90000830 (2B7) binding to the N-terminus of human HTT (amino acids 7–13) [16]; mouse monoclonal antibody CHDI-90000833 (4C9) binding against the poly-proline domain of human HTT (amino acids 51–71) [23]; S830 is a sheep polyclonal antibody that was raised against an exon 1 HTT protein with 53Q [27]. The mouse monoclonal antibody MAB2166, with the epitope mapped to amino acids 445–459 of the human HTT [28], was obtained from Millipore. The mouse monoclonal antibody CHDI-90000942 (MW8) was generated using soluble human GST-tagged exon 1-Q67 boosted with exon 1-Q67 aggregates; the epitope was mapped to amino acids 83–90 (AEEPLHRP) at the C-terminus of human exon 1 HTT [24]. SULFO-TAG (ST) labeling of antibodies 4C9, MW1, and MW8 was performed using the MSD SULFO-TAG NHS-Ester reagent (Meso Scale Discovery) according to manufacturers’ instructions.

### Recombinant huntingtin proteins

cDNA corresponding to the N-terminal 97 amino acids of human HTT including either 16 or 46 glutamine residues was cloned into a modified pTriEx-4 vector (Millipore) coding for an N-terminal MBP and C-terminal His6 tag. The 97 amino acid N-terminal sequence represents an extended version of the regular human exon 1 HTT sequence (amino acids 1–90) to include a short spacer between the MW8 epitope and the C-terminal His6 tag. The goal was to prevent the His6 tag from interfering with MW8 binding to HTT. The polyQ stretch was coded by a mixed CAG/CAA composition to reduce the risk of deletion or expansion during propagation and cloning. Proteins were expressed in *E*.*coli* BL21 (DE3). After lysis of cell pellets using a microfluidizer (Microfluidics), proteins were purified by sequential Ni-NTA and amylose affinity chromatography, followed by dialysis against a buffer containing 10 mM Tris (pH 8.0) and 100 mM NaCl. Proteins were concentrated using a 10 kDa MWCO Amicon Ultra spin concentrator (Millipore). Following determination of protein concentration, purified HTT protein (purity > 95%) was aliquoted and stored at -80°C. The HTT constructs are referred to as HTT(1–97)-Q16 and HTT(1–97)-Q46 throughout the publication. Human HTT(1–573)-Q23 and Q73 with N-terminal FLAG tag [19] and human full-length HTT-Q17 and Q46 [29] carrying an N-terminal FLAG and C-terminal His8 tag were provided by BioFocus, a Charles River Company (Leiden, NL) and stored at -80°C.

### Generation of HTT aggregates, aggregation kinetics, and inhibition of aggregation

The MBP-tag was introduced at the N-terminus of the HTT(1–97) constructs to maintain the proteins in a soluble state. The thrombin cleavage site inserted between the tag and HTT allows subsequent cleavage of the MBP-tag by thrombin protease, leading to the aggregation of HTT(1–97). For generation of aggregates, 10 μM MBP-tagged HTT(1–97)-Q16 or Q46 were mixed with 150 μg/mL bovine thrombin protease (Sigma) and 2 mM CaCl_2_ in a total volume of 200 μL 50 mM Tris (pH 8.0), 150 mM NaCl (aggregation buffer). Samples were incubated for 24 h at 37°C. Cleaved proteins were aliquoted, snap-frozen, and stored at -20°C and are stable for up to 6 months.

For monitoring aggregation kinetics, the same aggregation samples were used in a total volume of 400 μL. Aliquots were analyzed before and at multiple time points after initiation of aggregation by protease addition. Samples were immediately snap-frozen at each time point and stored at -20°C. Regarding concentrations of aggregated HTT(1–97)-Q46, assuming complete aggregation after 24 h incubation, the concentration of aggregates was set equal to the concentration of monomeric HTT before initiation of the aggregation process.

For inhibition of aggregation, protease cleavage and subsequent aggregation of 33 μM HTT(1–97)-Q46 was performed in the presence of different concentrations of the known HTT aggregation inhibiting peptide QBP1 and a scrambled (scr) QBP1 control [30]. Peptides carried an Antennapedia (Antp)-tag [31]. The isolated Antp-tag was also included as control. After 6 h of incubation at 37°C, samples were snap-frozen and stored at -20°C. For MSD analysis, samples were further diluted to 250 nM HTT(1–97)-Q46.

### Rodent models

Fresh snap-frozen whole brain samples from R6/2 mice strain B6CBA-Tg(HDexon 1)62Gpb/3J at 4, 6, 8, and 12 weeks of age (CAG repeat sizes (mean ± standard deviation): 4 weeks: 120 ± 2.3; 6 weeks: 123 ± 0.6; 8 weeks: 116 ± 3.2; 12 weeks: 114 ± 1.2), zQ175 knock-in mice strain C57BL/6J *Htt*^*tm1Mfc*^/190JChdi at 3, 6, 9, and 14 months of age (CAG repeat sizes (mean ± standard deviation): 3 months: 190 ± 6.0; 6 months: 187 ± 7.7; 9 months: 191 ± 3.0; 14 months: 186 ± 7.7), and BACHD mice strain C57BL/6 Tg(HTT*97Q)LXwy/JChdi at 3, 6, 9, and 12 months of age were (CAG repeat sizes stable at 97) obtained from the Jackson Laboratory (Bar Harbor, USA) and stored at -80°C until homogenization. Mice were euthanized by trained personnel by a lethal dose of carbon dioxide (CO_2_) followed by cervical dislocation prior to tissue collection. All procedures and protocols were run according to The Jackson Laboratory’s Institutional Animal Care and Use Committee (IACUC) Guidelines, and were conducted in compliance with the National Institutes of Health Guideline for Care and Use of Laboratory Animals.

Hemizygous R6/2 mice in the Bates Lab colony were maintained by backcrossing R6/2 males to (CBA/CaxC57BL/6J)F1 females (B6CBAF1/OlaHsd; Harlan Olac) and mice were genotyped and CAG repeat sized as previously described [22]. Mice were sacrificed by cervical dislocation. Cortex, striatum, and musculus tibialis anterior samples from R6/2 and wild type mice at 4, 8, and 14 weeks of age were dissected and snap frozen in liquid nitrogen. CAG repeat sizes (mean ± standard deviation) were as follows: 4 weeks (209 ± 3.9), 8 weeks (210 ± 2.4) and 14 weeks (210 ± 3.3). In the Bates Lab, all experimental procedures performed on mice were conducted under a license from the Home Office (Animal Scientific Procedures Act 1996) and approved by the Ethical Review Process Committee.

The R6/2 mouse model is transgenic for a genomic fragment spanning the 5’ end of the human *HTT* gene, including exon 1 [32, 33]. The zQ175 C57B/L6J knock-in mouse is derived from a spontaneous expansion of the CAG copy number in the originally described CAG 140 knock-in mouse model [34, 35]. The BACHD mouse model expresses full-length human mHTT with 97 glutamine repeats under the control of the endogenous HTT regulatory machinery on the bacterial artificial chromosome [26].

### AAV-ZFP in vivo application in an HD mouse model

Live zQ175 C57B/L6J knock-in mice and wildtype littermates were obtained from the Jackson Laboratory (Bar Harbor, USA). Two groups of 10 zQ175 mice received bilateral intra-striatal injections of viral particles encoding either the HTT allele-specific ZFP30640 or a control without the DNA-binding domain (ZFP-ΔDBD) [15] at 2 months of age. Mice were individually anaesthetized with 3% isoflurane at a flow rate of 1 L/min and placed in a small animal stereotaxic instrument (Kopf; Model No. 940). Anesthesia was maintained via gas nose cone delivery of 2% isoflurane at a flow rate of 0.5 L/min throughout the surgical procedure. The scalp of the animal was sterilized with 70% ethanol and iodine solution, followed by lidocaine application. A longitudinal mid-sagittal incision of 1 cm in length was made in the scalp. Following skin incision, a small hole corresponding to the striatal injection site was made in skull using an electrical drill (Foredom; Model No. H.30). The coordinates measured according to the mouse bregma were 0.8 mm anterior, 1.8 mm lateral on right and 3.8 mm deep from bregma with flat skull nosebar setting. A total volume of 4 μL (4E10 genome copies (GCs)) ZFP viral particles was administered using a Hamilton gas tight syringe (customized gauge 26 needle, model 1801 RN) connected to an automated microinjection pump with a constant flow rate of 200 nL/min. After injection, the surgery wound was sealed and the animals were kept on a heating pad until fully recovered. Mice were euthanized 4 months post-injection at 6 months of age. For subsequent MSD analysis, mice were sacrificed by decapitation after exposure to carbon dioxide, striata were prepared from removed whole brains, snap-frozen, and stored at -80°C until homogenization. All animal work was carried out in accordance with the regulations of the German animal welfare act and the EU legislation (EU directive 2010/63/EU). The study protocol was approved by the local Ethics committee of the Authority for Health and Consumer Protection of the city and state of Hamburg (“*Behörde für Gesundheit und Verbraucherschutz”* BGV, Hamburg) under the file number #V11307/591 00.33.

### Tissue homogenization

Whole brain homogenates were lysed using a T25 ULTRA-TURRAX (IKA) with three 30 s pulses in 5 mL tissue lysis buffer containing 20 mM Tris (pH 7.5), 150 mM NaCl, 1 mM EDTA, 1 mM EGTA, 1% Triton X-100, 10 mM NaF, 1 mM PMSF, Phosphatase Inhibitor Cocktail II (Sigma), Phosphatase Inhibitor Cocktail III (Sigma), Protease Inhibitors (Roche Diagnostics). Crude lysates were centrifuged three consecutive times for 12 min at 3,000 x g and 4°C, the supernatant was transferred to a new tube after each centrifugation step. Dissected cortex, striatum and musculus tibialis anterior samples were homogenized in 250–400 μL tissue lysis buffer using lysing matrix M tubes (MP Biomedicals), striatal samples of ZFP-treated mice were homogenized in 80 μL of tissue lysis buffer using Precellys CK14 lysing tubes (Berlin Technologies) in a FastPrep-24 tissue homogenizer (MP Biomedicals) with three 30 s cycles. Crude lysates were centrifuged three consecutive times for 10 min at 16,000 rcf and 4°C, and the supernatant was transferred to a new tube after each centrifugation step. For all tissue homogenates, the total protein concentration was determined using the bicinchoninic acid assay (BCA; Thermo Scientific) and adjusted to 1.5, 2, or 5 mg/mL with tissue lysis buffer. Homogenates were aliquoted, snap-frozen, and stored at -80°C.

### Size-exclusion chromatography (SEC)

500 μL of 3–5 mg/mL whole brain homogenates from a heterozygous zQ175 (age: 14 months) or R6/2 mouse (age: 12 weeks) were fractionated by SEC on a Superose 6 10/300 GL column (GE Healthcare Life Sciences). For calibration of the column, 500 μL of 5 fmol/μL full-length HTT-Q17 and Q46, 25 fmol/μL of MBP-tagged HTT(1–97)-Q46, and 200 fmol/μL thrombin-digested HTT(1–97)-Q46 were included. All SEC experiments were performed in SEC buffer (20 mM Tris pH 7.5, 150 mM NaCl, 1 mM EGTA, 1 mM EDTA, 1% Triton X-100) at 4°C with a flow rate of 0.5 mL/min, and elution of 1.2 column volume. Fractions (300 μL volume/fraction) were collected in a 96-well plate and diluted 1:1 in blocking buffer (2% probumin/0.2% Tween-20 in PBS) for MSD analysis.

### MSD assays

MSD 384-well plates (Meso Scale Discovery) were coated overnight at 4°C with 10 μL of coating antibody in a carbonate-bicarbonate coating buffer (15 mM Na_2_CO_3_/35 mM NaHCO_3_, pH 9.6) per well. Plates were then washed three times with 35 μL of wash buffer (0.2% Tween-20 in PBS) per well and blocked with 35 μL of blocking buffer (2% probumin/0.2% Tween-20 in PBS) per well for 1 h at RT with rotational shaking. Recombinant proteins or brain extracts were diluted to the desired concentrations in a mixture of aggregation buffer or tissue lysis buffer and at least 50% blocking buffer. For spike recovery experiments, recombinant protein samples were spiked into 1 mg/mL of a wildtype zQ175 mouse whole brain lysate. After an additional washing step, 10 μL per sample was transferred to each well of the antibody-coated MSD plate and incubated with shaking for 1 h at RT. After disposal of samples and four wash cycles with 35 μL of wash buffer each, 10 μL of the primary detection antibody (SULFO-TAG (ST) labeled or non-labeled) was added to each well and incubated with shaking for 1 h at RT. For non-labelled primary detection antibodies, 10 μL goat anti-mouse SULFO-TAG labeled secondary detection antibodies (1:1,000 in blocking buffer; Meso Scale Discovery) was added to each well after an additional washing step and incubated with shaking for 1 h at RT. After washing three times with wash buffer, 35 μL of read buffer T with surfactant (Meso Scale Discovery) was added to each well and the plate was imaged on a Sector Imager 6000 (Meso Scale Discovery) according to manufacturers’ instructions. The following antibody combinations were used: 4 μg/mL pAb147 (coating) / 1:1,000 MAB2166 (detection) / 1:1,000 anti-mouse-ST (secondary detection); 5 μ = g/mL 2B7 / 0.1 μg/mL 4C9-ST; 5 μg/mL 2B7 / 5 μg/mL MW1-ST; 4 μg/mL MW8 / 1 μg/mL 4C9-ST; 2 μg/mL MW8 / 5 μg/mL MW8-ST. Optimal antibody concentrations may vary with each new ST-labelled antibody batch.

### TR-FRET assays

The samples tested in the MSD assays were also applied for TR-FRET analysis. 5 μL per sample in tissue lysis buffer and at least 50% blocking buffer was transferred to a low-volume 384-well plate (Greiner), followed by addition of 1 μL of antibody mix in 50 mM NaH_2_PO_4_, 400 mM NaF, 0.1% BSA, 0.05% Tween20 and incubation for 1 h at RT. Samples were measured using an EnVision plate reader (PerkinElmer) with excitation at 320 nm and readout at 615 nm and 665 nm (d2) or 530 nm (AlexaFluor 488), respectively. Used antibody combinations and final amounts per well comprise: 0.25 ng 2B7-Tb / 20 ng MW1-d2; 1 ng 4C9-Tb / 10 ng 4C9-AlexaFluor 488 (AF488); 1 ng MW8-Tb / 10 ng MW8-d2. Antibody labeling with Tb, d2, or AF488 was performed by Cisbio Bioassays (Codelet, France).

### Seprion ELISA

2.5% w/v lysates were prepared in RIPA buffer (50 mM Tris/HCl pH 8.0, 150 mM NaCl, 1% Igepal CA-630, 0.5% sodium deoxycholate, 0.1% SDS, 1 mM β-mercaptoethanol, 1 mM PMSF, 0.5 mM DTT, and mini-protease inhibitor cocktail (Roche Diagnostics)) by ribolysing in lysis matrix D tubes (MP Biomedicals) 2 times for 30 s at 4°C, transferred to ice for 5 min for cooling before the third 30 s ribolysation step. Lysates were centrifuged at 13,000 rpm for 2 min. Supernatants were used immediately or snap-frozen on dry ice and stored at -80°C. 15 μL of the 2.5% ribolysate was mixed with 3 μL 10% SDS, 62 μL water and 20 μL of 5x Microsens capture buffer in PCR tubes. The sample was transferred to a Seprion ELISA plate (cat# SEP1-96-01; Microsens Biotechnologies) and incubated for 1 h at 23°C in a plate shaker. Wells were washed 5 times with 0.1% PBS-T (PBS / 0.1% Tween-20) and any residual buffer was removed. 100 μL of the primary antibodies S830 or MW8 were added at 1:2,000 dilution in conjugate buffer (150 mM NaCl, 4% BSA (98% electrophoretic grade), 0.1% Tween 20, 1% skimmed milk powder) and incubated at 23°C for 1 h. The washing step with PBS-T was repeated 5 times, followed by incubation with 100 μL of an HRP-conjugated anti-sheep (Abcam) or anti-mouse secondary antibody (ThermoFisher) at 1:2,000 dilution in conjugate buffer for 45 min at 23°C. The washing step with PBS-T was repeated 5 times and 100 μL TMB substrate (warmed to 23°C) was added. The reaction was stopped with 100 μL of 0.5 M HCl when the required saturation was reached, and absorption was read at 450 nm using a Bio-Rad plate reader.

## Results

### Detection of recombinant HTT aggregates

The MW8 antibody, known to bind aggregated HTT [24], was used in the development of the MSD-based HTT aggregate assays. As a suitable standard protein, a maltose binding protein (MBP)-tagged human HTT fragment (aa 1–97 to include the MW8 epitope and a short spacer before the C-terminal His6 tag) with a polyQ stretch of 46 glutamines (HTT(1–97)-Q46) was generated. The MBP-tag was incorporated to maintain the protein in a soluble monomeric state. Thrombin protease-cleavage of the MBP-tag and incubation at 37°C induces aggregation [36]. First, we compared concentration response curves for HTT(1–97)-Q46 before and after 24 h of aggregation. In these experiments, an equivalent HTT(1–97)-Q16 control protein containing a non-pathogenic polyglutamine domain was included which does not form aggregates after protease digestion in comparison to the mutant HTT fragment. We confirmed almost complete cleavage of the MBP-tag after 24 hours incubation with the protease by western blotting for both constructs. In addition, the observed band for HTT(1–97)-Q46 was less prominent than the one for HTT(1–97)-Q16, reflecting the incorporation of digested HTT(1–97)-Q46 into larger aggregates detected at a higher molecular weight band (S1 Fig).

**Fig 1:**
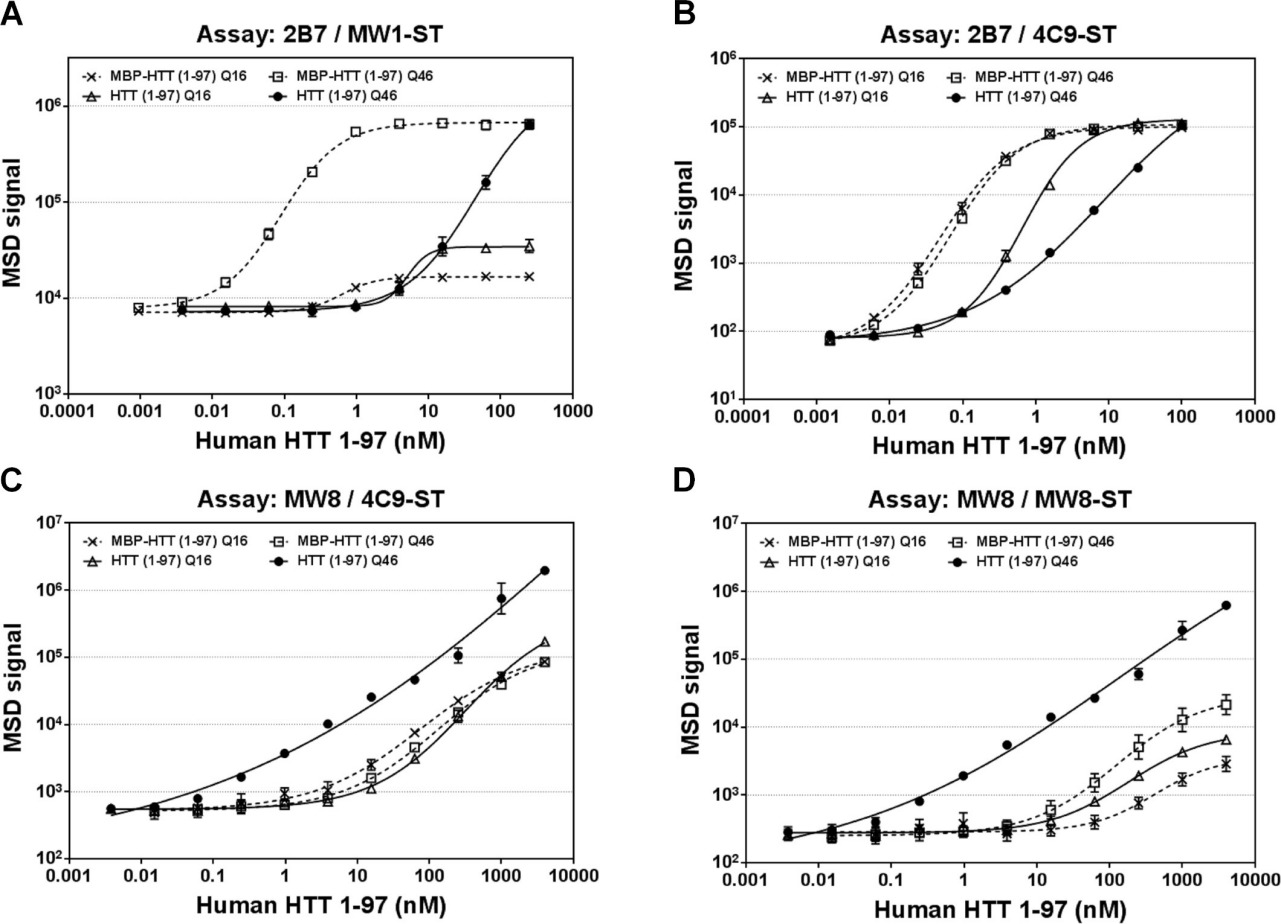
Monomer and aggregate assays for analysis of HTT(1–97)-Q16 and Q46. MBP-tagged and thrombin-digested human HTT(1–97)-Q16 and Q46 were tested after 24 h incubation at different concentrations in the monomer assays: 2B7 / MW1-ST (A) and 2B7 / 4C9-ST (B), and the aggregate assays: MW8 / 4C9-ST (C) and MW8 / MW8-ST (D). Error bars represent standard deviation for three independent measurements. In some cases, error bars are too small to be visible.

The MBP-tagged and thrombin-digested HTT(1–97)-Q16 and HTT(1–97)-Q46 were first analyzed in the 2B7 / MW1-ST and 2B7 / 4C9-ST assays (S2 Fig). Because the 2B7 / MW1-ST assay is selective for polyQ expanded monomeric human HTT, no to minimal signal was detected for HTT(1–97)-Q16, while detection of HTT(1–97)-Q46 was significantly reduced after thrombin digestion (Fig 1A). Analysis of the MBP-tagged HTT(1–97) constructs in the 2B7 / 4C9-ST assay for total, polyQ-independent, monomeric human HTT showed similar results to those obtained with the 2B7 / MW1-ST assay: detection of HTT(1–97)-Q46 was strongly reduced after cleavage of the MBP-tag. A small reduction in detection was also observed for HTT(1–97)-Q16 (Fig 1B). In summary, the results indicate that detection of HTT(1–97)-Q46 monomer levels was reduced due to aggregation after cleavage of the MBP-tag. Signal detection in the thrombin-digested samples was still noticeable, likely due to epitope availability on the aggregate surface. However, the shift in the response curve for digested HTT(1–97)-Q16 in the 2B7 / 4C9-ST assay could also reflect an oligomerization or aggregation process of the shorter polyQ domain, indicating that incubation at high concentrations for 24 h at 37°C had induced oligomerization of HTT(1–97)-Q16 [37].

For the development of potential aggregation assays, multiple combinations of MW8 with other anti-HTT antibodies were tested for their ability to detect a signal increase after protease digest and subsequent incubation. MW8 was evaluated as a capture antibody, as well as a detection antibody. Best results were obtained for the following antibody combinations: MW8 / 4C9-ST and MW8 / MW8-ST. Both putative MSD aggregate assays showed the strongest signal response for aggregated HTT(1–97)-Q46 (Fig 1C and 1D) with a lower limit of detection (LLOD) of approximately 10 pM and a linear range across at least 4 log units (Table 1). Importantly, both MBP-tagged monomeric HTT(1–97) constructs and thrombin-digested HTT(1–97)-Q16 were detected to a significantly lower extent, with LLODs for these constructs being at least 100-fold higher than for aggregated HTT(1–97)-Q46 (Table 1).

**Table 1:** HTT assay sensitivity and dynamic range.

	MSD assay MW8 / 4C9-ST	MSD assay MW8 / MW8-ST	TR-FRET assay MW8-Tb / MW8-d2	TR-FRET assay 4C9-Tb / 4C9-AF488
**LLOD;** **MBP-HTT(1–97)-Q16**	0.8 nM	19 nM	-	-
**LLOD; Digested HTT(1–97)-Q16**	1.3 nM	2.7 nM	-	-
**LLOD; MBP-HTT(1–97)-Q46**	0.9 nM	1.8 nM	-	-
**LLOD; Digested HTT(1–97)-Q46**	8 pM	18 pM	38 nM	18 nM
**Fold difference of LLOD; HTT(1–97)-Q46 before and after thrombin digest**	~ 100x	~ 100x	-	-
**Fold difference of LLOD; HTT(1–97)-Q16 and Q46 after thrombin digest**	~ 150x	~ 150x		
**Dynamic range (log units)**	~ 4	~ 4.5	~ 2	~ 2

Lower limit of detection (LLOD) was determined using the Discovery Workbench 4.0 software (Meso Scale Discovery) using the mean of the background plus 2.5 times the standard deviation. Log units were defined by visual evaluation.

In summary, assays 2B7 / MW1-ST and 2B7 / 4C9-ST showed a clear preference for detection of HTT monomers (Fig 1A and 1B), whereas assays MW8 / 4C9-ST and MW8 / MW8-ST preferentially detected HTT protein at higher aggregation states (Fig 1C and 1D). Detection of aggregated HTT(1–97)-Q46 was also confirmed by immunoprecipitation with MW8 (S3 Fig). However, our data also suggests that the first two assays detected aggregated HTT to some extent, while the latter ones also showed detection of a weaker signal response for non-aggregated HTT. Nonetheless, to facilitate matters, we will refer to assays 2B7 / 4C9-ST and 2B7 / MW1-ST as “monomer assays” and to assays MW8 / 4C9-ST and MW8 / MW8-ST as “aggregate assays” herein.

**Fig 2:**
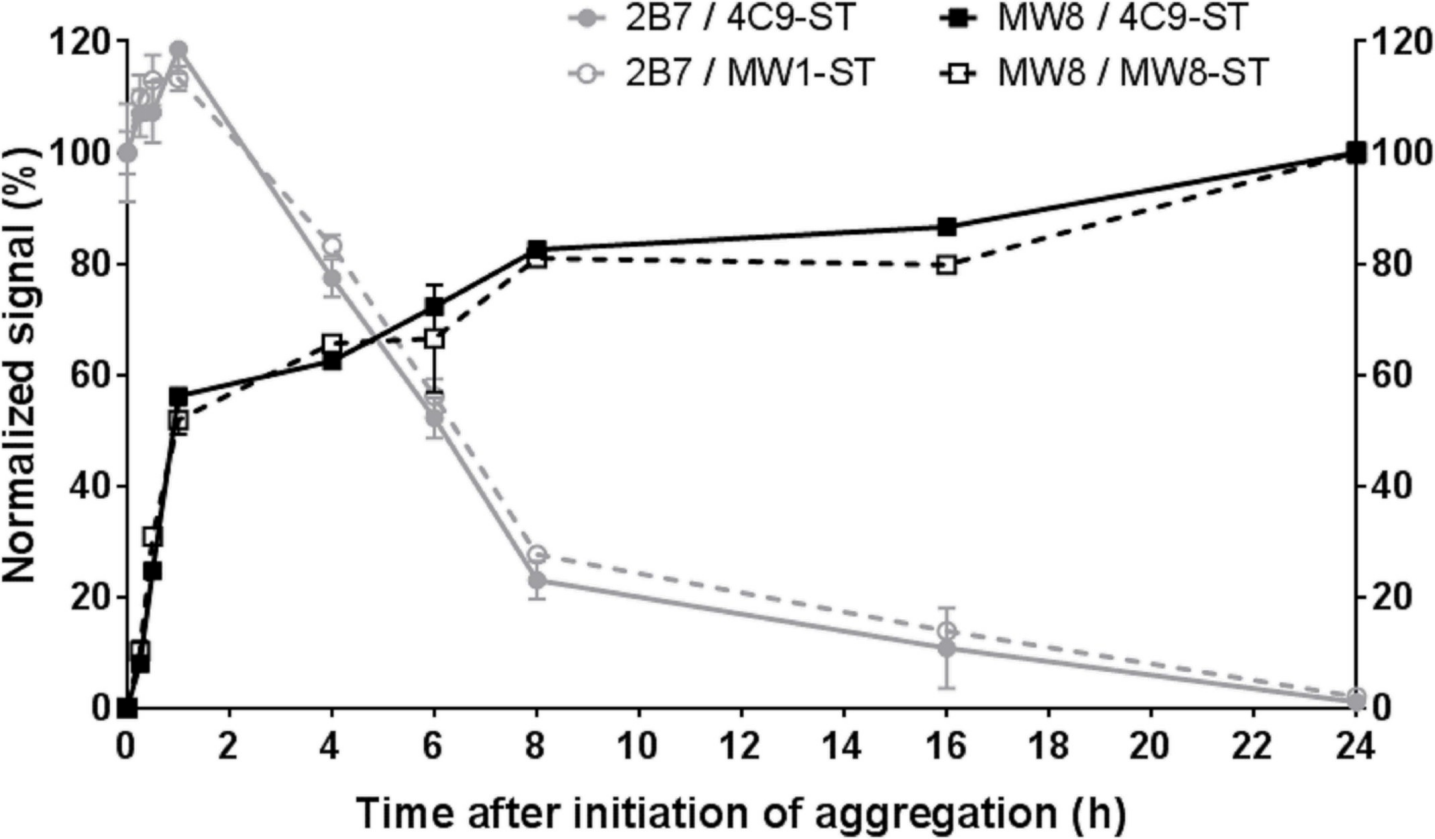
Aggregation kinetics of HTT(1–97)-Q46. Aggregation of HTT(1–97)-Q46 was initiated by addition of thrombin protease, followed by incubation for 24 h at 37°C. Samples were taken at the given time points and analyzed at 1 nM in the 2B7 / 4C9-ST and 2B7 / MW1-ST assays for monomeric human HTT or human mHTT, respectively. Additionally, samples were measured at 1 μM in the MW8 / 4C9-ST and MW8 / MW8-ST aggregate assays. Data were normalized: signal at 0 h was set to 100% for both 2B7 assays; signal at 24 h was set to 100% for both MW8 assays. Results were confirmed in three independent runs with one representative dataset shown. Error bars represent standard deviation for three technical replicates. In some cases, error bars are too small to be visible.

When monitoring aggregation kinetics, a gradual signal increase was observed over time with a plateau after approximately 8 h for the antibody combinations MW8 / 4C9-ST and MW8 / MW8-ST (Fig 2). Aggregation kinetics using the MW8 antibody were also confirmed by an orthogonal filter retardation assay (S4 Fig). Simultaneously, a time-dependent signal decrease was measured in the MSD assays 2B7 / 4C9-ST for total human HTT and 2B7 / MW1-ST for human mHTT (Fig 2).

**Fig 3:**
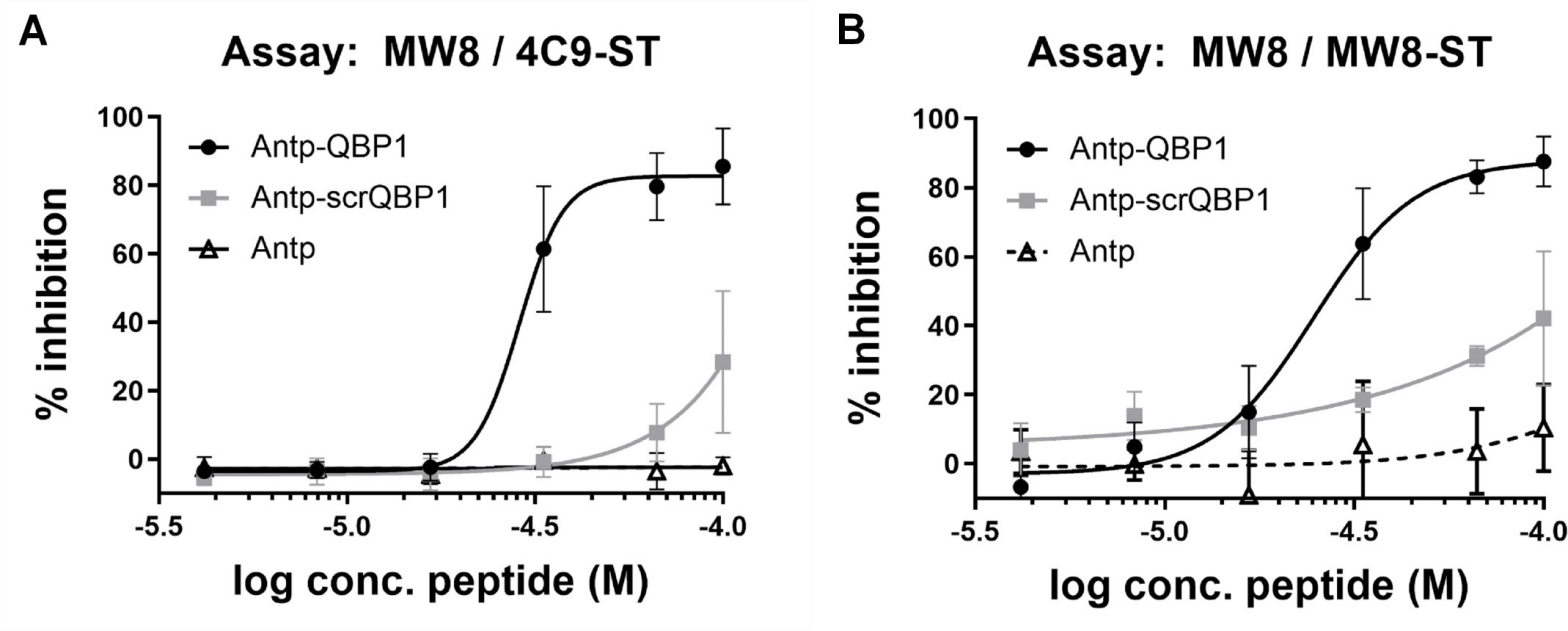
Inhibition of aggregation. Protease-cleavage and subsequent aggregation of 33 μM MBP-HTT(1–97)-Q46 were performed in presence of a concentration series of peptides Antennapedia (Antp), Antp-QBP1, and Antp-scrQBP1. After aggregation, samples were diluted to 250 nM HTT(1–97)-Q46 and analyzed in the MW8 / 4C9-ST (A) and MW8 / MW8-ST assays (B). Error bars represent standard deviation for three independent measurements.

It has been previously shown that the peptide QBP1 is capable of preventing proteins with polyQ stretches from aggregating [30, 31]. We therefore performed a thrombin-digest with subsequent incubation for 24 h at 37°C in the presence of Antp-QBP1, with negative controls Antp-scrQBP1 and Antp, to see if treatment reduced the signals detected for aggregated HTT(1–97)-Q46 by blocking aggregate formation. As detected in the MW8 / 4C9-ST (Fig 3A) and MW8 / MW8-ST (Fig 3B) assays, Antp-QBP1 indeed inhibited aggregation with apparent IC_50_ values of 28.7 μM (MW8 / 4C9-ST) and 24.8 μM (MW8 / MW8-ST). The Antp-scrQBP1 showed a minor inhibitory effect but only at IC_50_ values above the highest tested peptide concentration of 100 μM. Antp did not show any inhibitory effect. These data are consistent with the HTT proteins being in an aggregated state in the two assays.

To demonstrate that the newly developed assays specifically detect HTT aggregate conformations, we examined whether the assays could detect amyloid aggregates as found in the brain of Alzheimer’s patients [38]. Amyloid- β (Aβ) fibrils were generated from Aβ42 peptides *in vitro* and aggregation was confirmed by Thioflavin T assay (S5A Fig). Analysis of Aβ fibrils in the assays MW8 / 4C9-ST and MW8 / MW8-ST resulted in an absence of signal detection (S5B and S5C Fig). This indicates that the two HTT aggregate assays are not general pan-aggregate assays as they show selectivity for HTT aggregates when compared to Aβ fibrils generated *in vitro*.

In addition to HD there are other CAG repeat disorders such as Machado-Joseph Disease (MJD), also known as spinocerebellar ataxia type 3 (SCA3). In MJD, the disease is caused by an abnormal CAG expansion in the gene for ATXN3 [39]. We therefore tested our assays to see if they would detect any polyQ-dependent aggregates independent of the surrounding sequences. To this end, we used a GST-tagged polyQ46 protein which was aggregated after thrombin-cleavage of the GST-tag. PolyQ46 aggregation was confirmed by Thioflavin T assay (S5D Fig). In the following MSD analysis, no signals were detected for aggregated polyQ46 in both the MW8 / 4C9-ST or the MW8 / MW8-ST assays (S5E and S5F Fig). This data demonstrates that our assays do not detect any polyQ aggregate but rather are specific for aggregated mHTT.

Before applying the novel HTT aggregate assays to the analysis of tissue samples, the recovery rate for spiked aggregated HTT(1–97)-Q46 standard curves in 1 mg/mL whole brain lysate from wild type mice were measured. None of the assays were significantly affected by the tissue matrix (S6 Fig).

### Detection of native aggregates from HD mouse models

**Fig 4:**
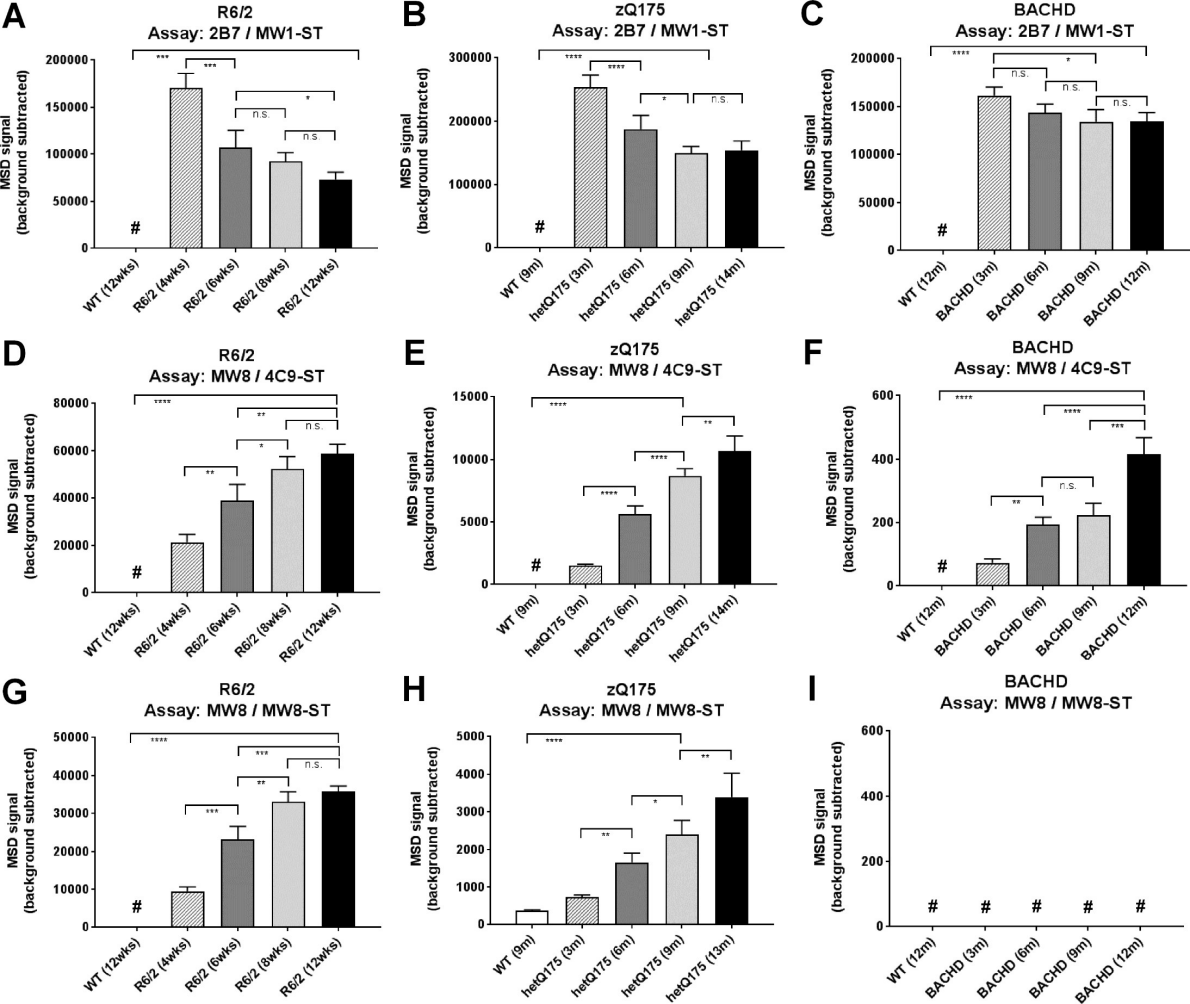
Analysis of human mHTT and HTT aggregates in an age series of HD mouse models. Whole brain lysates of an age series of R6/2 (left), hetQ175 (middle), and BACHD (right) mice were analyzed at 0.5 mg/mL (R6/2 and hetQ175), 0.1 mg/mL (BACHD, mHTT), and 2.5 mg/mL (BACHD, aggregates) in the 2B7 / MW1-ST (A-C) assay for human mHTT and the MW8 / 4C9-ST (D-F) and MW8 / MW8-ST (G-I) assay for aggregated HTT. Background: signal generated by sample buffer. Error bars represent standard deviations of at least three biological replicates. Samples were compared based on a one-way ANOVA statistical analysis: * p < 0.05, ** p < 0.01, *** p < 0.001, **** p < 0.0001, n.s. not significant. # below limit of detection.

Following identification of putative HTT MSD assays to detect recombinant HTT aggregates, we next tested these assays for detection of age-dependent native HTT aggregates formed in the brain of three different HD mouse models. It has been previously shown by immunohistochemistry (IHC) that the amount of HTT aggregates increases with age in R6/2 [19, 22], zQ175 [13], and BACHD [26] mice. In general, endogenous mouse HTT levels determined in the pAb147 / 2166 / anti-mouse-ST assay [19] were constant across different ages and between wild type and respective HD animals for R6/2 and BACHD mice (S7A and S7C Fig). However, while analysis of whole brain lysates from 3 and 14 month-old heterozygous Q175 (hetQ175) mice showed comparable levels of mouse HTT, it is noted that WT mice showed approximately double the signal intensity due to the human sequence knock-in construct of the hetQ175 animals not being detected by the mouse MSD assay (S7B Fig). Signals detected by the mHTT-specific 2B7 / MW1-ST assay decreased with age in all three HD models, potentially due to the loss of monomeric mHTT by incorporation into aggregates (Fig 4A–4C). Vice versa, analysis of tissue homogenates in the two aggregate assays, MW8 / 4C9-ST and MW8 / MW8-ST, showed an age-dependent increase in assay signals (Fig 4D–4I). Strongest signals were detected in R6/2 animals, followed by hetQ175 mice. Signals were observed to be low for BACHD animals in the MW8 / 4C9-ST assay (Fig 4F) and below the limit of detection in the MW8 / MW8-ST assay (Fig 4I). These results could potentially reflect the aggregate load in the various HD mouse models. The exon 1 construct in R6/2 animals shows the strongest tendency for aggregation. The full-length HTT construct with humanized exon 1 (~190Q) in zQ175 mice (or more likely an exon1 HTT protein generated through incomplete splicing of the full-length HTT sequence [40]) shows a significant level of aggregation, but clearly to a lower extent in comparison to R6/2 animals. Only a small amount of aggregates can be detected for human full-length HTT (97Q) in the BACHD mice, concomitant with the smallest age-dependent decrease of soluble mHTT levels of all three models (Fig 4C). Overall, the MSD data for the HD mouse model age series confirms the previously published IHC data, and therefore supports the preferential detection of aggregates in the newly developed MSD assays.

As shown in Fig 4 and S6 Fig, multiple HTT species are present in brain tissue homogenates from HD mouse models: endogenous non-expanded mouse HTT, genetically introduced human exon 1 or full-length mHTT, and HTT aggregates. To confirm that the newly developed assays were selective for aggregate species present in these samples, whole brain homogenates from a 12 week-old R6/2 and a 14 month-old hetQ175 mouse were separated on a Superose 6 size-exclusion chromatography (SEC) column followed by analysis of each elution fraction. In this procedure, proteins are separated by size, with the larger size proteins found in the earlier elution fractions.

The SEC column was initially calibrated using several recombinant HTT proteins: MBP-tagged monomeric HTT(1–97)-Q46 (Mw: 58 kDa), thrombin-digested, aggregated HTT(1–97)-Q46, human full-length HTT-Q17 (Mw: 347 kDa) and Q46 (Mw: 351 kDa). The main elution peak for HTT(1–97)-Q46 monomers was detected at 16 mL (S8A Fig), while the aggregated HTT(1–97)-Q46 eluted at approximately 8 mL (S8B Fig). Both full-length HTT-Q17 and Q46 eluted at 11–12 mL (S8C and S8D Fig). Elution peaks in the tissue homogenate samples were identified by comparison to these standard peaks.

**Fig 5:**
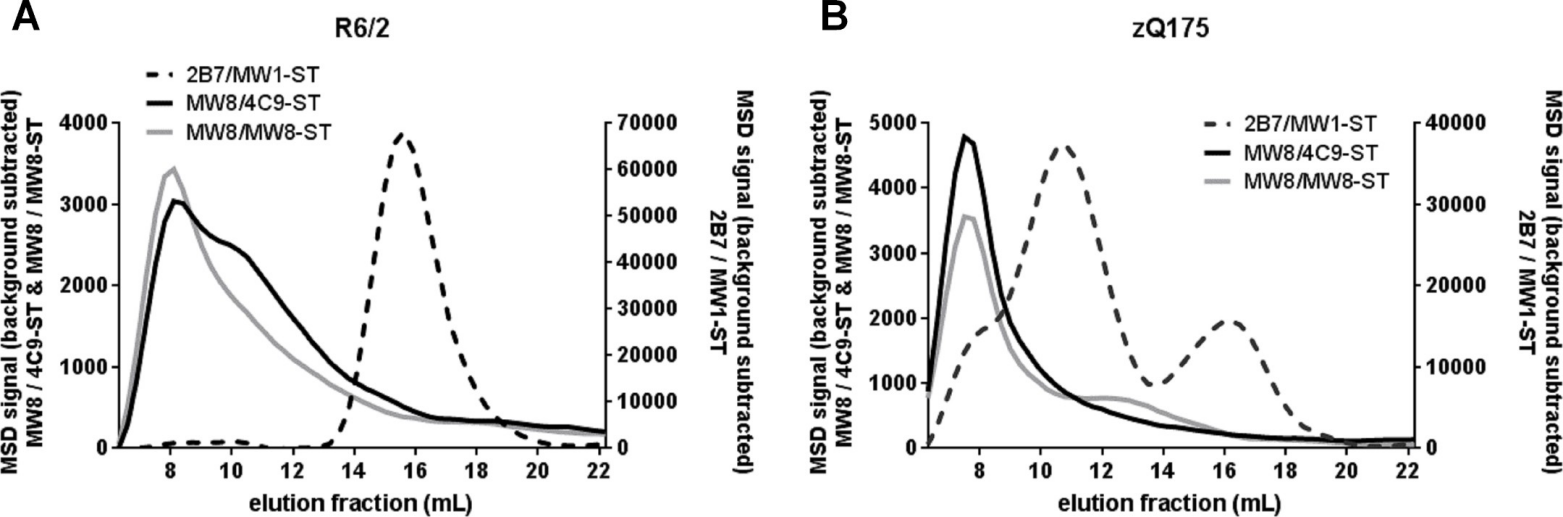
Analysis of R6/2 and zQ175 whole brain lysates after SEC. Whole brain homogenates from (A) a 12 week-old R6/2 (500 μL at 3 mg/mL) and (B) a 14 month-old hetQ175 mouse (500 μL at 5 mg/mL) were run on a Superose 6 SEC column. Elution fractions were subsequently analyzed in the 2B7 / MW1-ST assay for human mHTT and the aggregate assays MW8 / 4C9-ST and MW8 / MW8-ST. Background: signal generated by sample buffer. Curve shapes were smoothed. Confirmation of detected HTT species by Western blotting of individual elution fractions failed as samples were too dilute after SEC.

The elution profile obtained for the R6/2 whole brain lysate (Fig 5A) showed one single peak in the 2B7 / MW1-ST monomer assay at 16 mL of elution, reflecting monomeric exon 1. In the putative aggregate-selective MW8 / 4C9-ST and MW8 / MW8-ST assays, the major signal was detected at 8 mL of elution, corresponding to that of recombinant HTT(1–97)-Q46 aggregates. The potential aggregate elution peaks in both assays were relatively broad. The corresponding size of the HTT species detected in these fractions was larger than the expanded exon 1 monomer expressed in R6/2 mice, and could therefore represent a wide range of aggregate sizes.

The elution profile of the hetQ175 whole brain lysate (Fig 5B) showed one single peak at 8 mL of elution in the MW8 / 4C9-ST and MW8 / MW8-ST assays, also in line with the recombinant aggregate standard, and showing a comparable profile to that detected in the R6/2 mice. Two elution peaks were observed in the 2B7 / MW1-ST monomer assay: a larger elution peak at 12 mL and a smaller peak at 16 mL. HetQ175 mice express full-length mHTT reflected by the larger peak at 12 mL. Comparison with the elution profile of an MPB-HTT(1–97)-Q46 standard (S8A Fig) suggests that the minor peak detected at 16 mL most likely represents monomeric exon 1 HTT, or potentially also smaller N-terminal mHTT fragments that cannot be resolved by the applied SEC method. It has previously been shown that in zQ175 mice, and other knock-in models, exon 1 does not always splice to exon 2, thereby generating a small polyadenylated mRNA that encodes the mutant exon 1 HTT protein [40]. The amount of soluble exon 1 HTT decreases in zQ175 brains with disease progression [41]. In summary, elution peaks containing the major signal for aggregated HTT were observed for both mouse models by both aggregate assays, and were in accordance with peaks observed for recombinant HTT samples. Overall, analysis of lysates from HD model mouse brains after SEC supported the detection of HTT aggregates in both assays.

**Fig 6:**
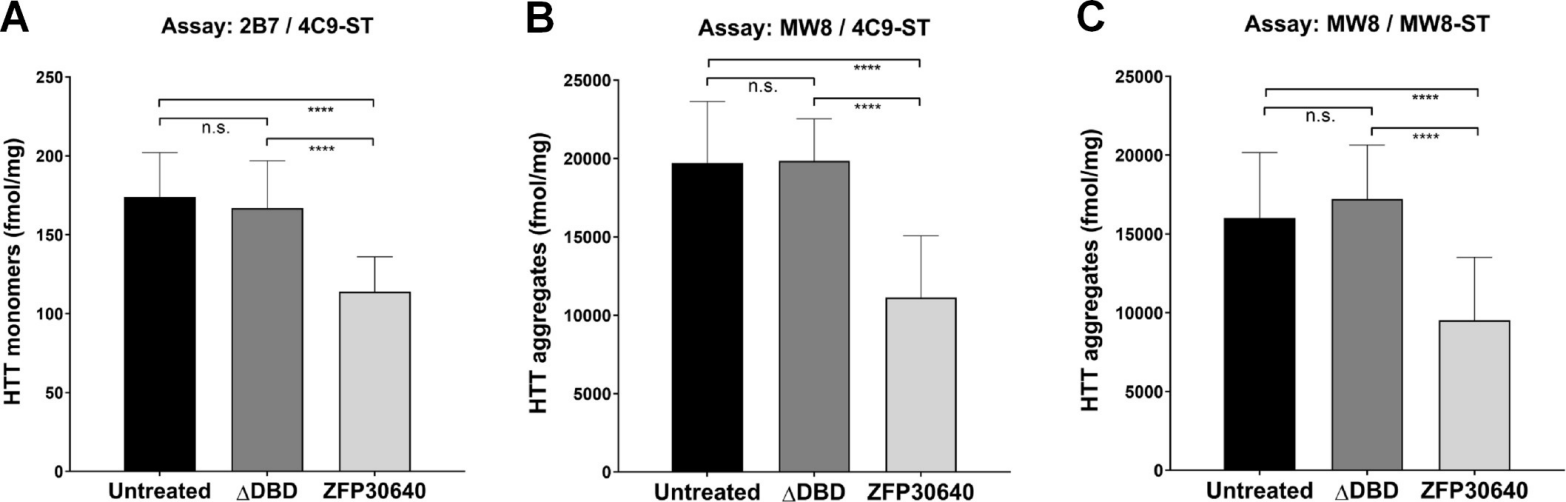
MSD analysis of AAV-ZFP-treated zQ175 mice showed reduction of mutant HTT and HTT aggregate levels. Quantification of human mHTT (A) or HTT aggregates (B and C). Analysis of striatal tissues from untreated hetQ175 mice, animals injected with AAV-SWB-ZFP-ΔDBD, or AAV-SWB-ZFP30640. Error bars represent standard deviation for analysis of at least 20 samples. n.s. not significant, **** p < 0.0001.

We next examined the ability of the MSD assays to detect changes in HTT aggregate levels in brain tissue following zinc-finger mediated repression of mutant *Htt* gene expression. In brief, a transcription repressor protein was directed via a zinc-finger domain to the CAG tract of the *Htt* gene. The zinc-finger domain was designed to specifically target the mutant form of the gene. Subsequently transcription of mHTT was blocked. Due to a marked reduction in the level of polyQ-expanded HTT monomers, ZFP treatment leads to a subsequent reduction in HTT aggregate formation [14, 15]. Based on these observations, intra-striatal injection of AAV particles carrying either a FLAG-tagged ZFP construct (AAV-ZFP-30640; S9A Fig) or a negative control ZFP construct lacking the DNA-binding domain (AAV-ZFP-ΔDBD; S9B Fig) was performed in two month-old hetQ175 mice. Four months after treatment, mice were sacrificed and brain tissue was collected. IHC analysis of whole brain sections from treated mice was performed with an anti-FLAG antibody. Results indicated that the AAV injection transduced more than 50% of the striatum (S9C and S9D Fig). In AAV-ZFP-ΔDBD treated animals, HTT aggregates detected with the EM48 antibody were abundant throughout the striatum. In contrast, aggregate levels were reduced by more than 90% in FLAG-positive medium spiny neurons of AAV-ZFP-30640 treated mice (S9C–S9F Fig). For subsequent testing in the aggregate-selective MSD assays, striata from AAV-ZFP-30640 and AAV-ZFP-ΔDBD treated animals, as well as untreated control mice, were dissected and homogenized. Analysis of striatal lysates in both the 2B7 / 4C9-ST assay for total monomeric human HTT and the MW8 / 4C9-ST and MW8 / MW8-ST aggregate assays showed a 30–50% reduction in HTT levels in AAV-ZFP-30640 treated samples compared to AAV-ZFP-ΔDBD and untreated samples (Fig 6). Quantification of HTT aggregate levels showed comparable results for both aggregate assays, with levels around 15,000–20,000 fmol/mg for the untreated and AAV-ZFP-ΔDBD samples. While data from the MSD assays confirmed the IHC results, the calculated 30–50% reduction in HTT aggregates was significantly lower than the 90% reduction of both nuclear and perinuclear aggregates observed by IHC. This difference can be explained by the fact that only AAV-infected medium spiny neurons were evaluated in the IHC analysis, while the whole striatum, including non AAV-transduced areas, was used for analysis in the MSD assays.

### Comparison with existing assay technologies

**Fig 7:**
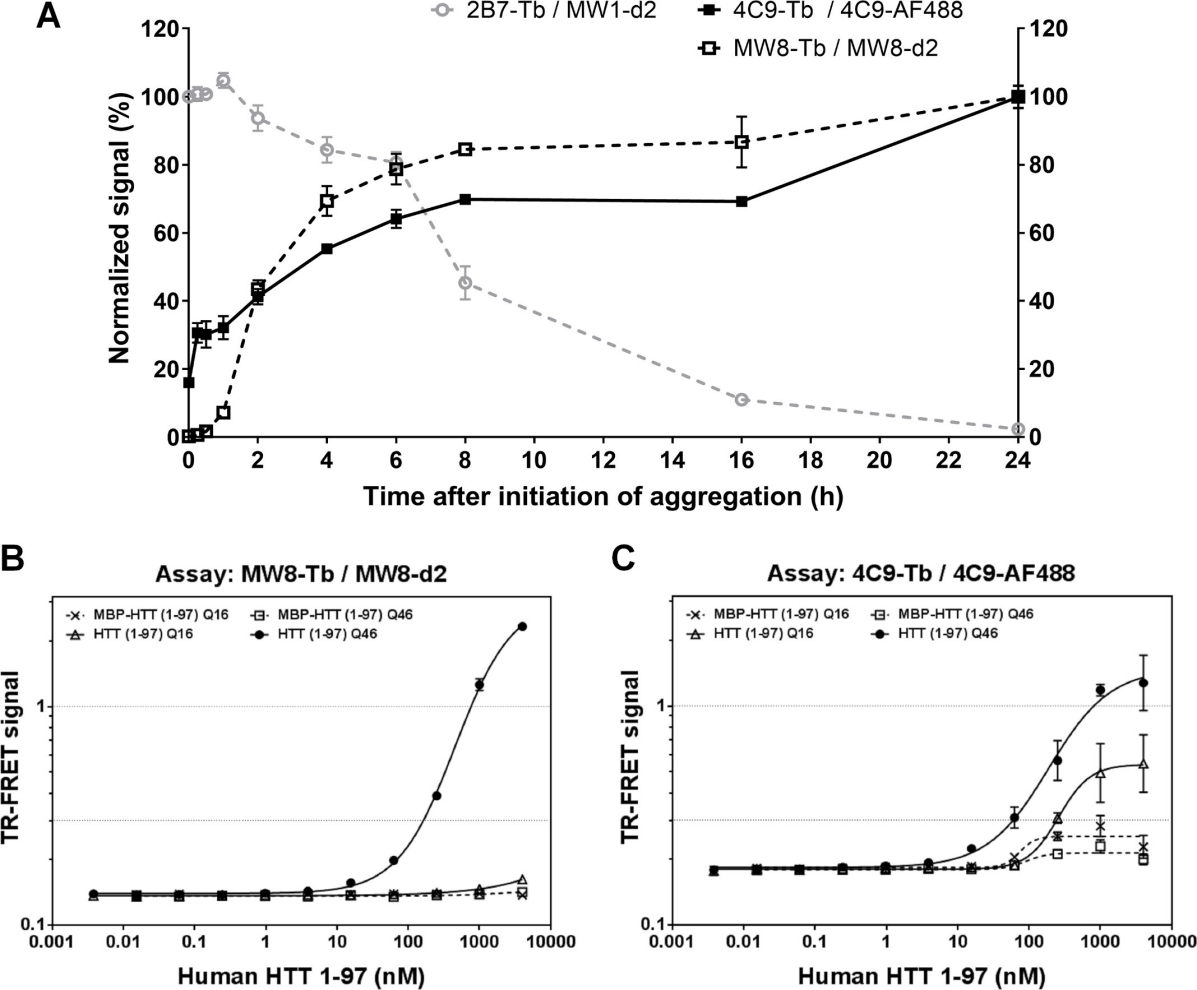
Analysis of HTT(1–97)-Q16 and Q46 by TR-FRET. (A) Aggregation kinetics of HTT(1–97)-Q46. Aggregation of HTT(1–97)-Q46 was initiated by addition of thrombin protease, followed by incubation for 24 h at 37°C. Samples were taken at the given time points and analyzed at 1 nM in the 2B7-Tb / MW1-d2 TR-FRET assay for human mHTT. Additionally, samples were measured at 1 μM in the MW8-Tb / MW8-d2 and 4C9-Tb / 4C9-AF488 TR-FRET assays for HTT aggregates. Data was normalized: signal at 0 h was set to 100% for the 2B7-Tb / MW1-d2 assay; signal at 24 h was set to 100% for the MW8-Tb / MW8-d2 and 4C9-Tb / 4C9-AF488 assays. Results were confirmed in three independent runs with one representative dataset shown. Error bars represent standard deviation for three technical replicates. (B) and (C) Analysis of MBP-tagged and thrombin-digested human HTT 1–97 Q16 and Q46 at different concentrations in the MW8-Tb / MW8-d2 (B) and 4C9-Tb / 4C9-AF488 (C) TR-FRET aggregate assays. Error bars represent standard deviation for three independent measurements. In some cases, error bars are too small to be visible.

Two different assay systems have been previously described for the detection of HTT aggregates: 1) TR-FRET assay with the antibody combinations 4C9-Tb / 4C9-AF488 and MW8-Tb / MW8-d2 [23]; and 2) Seprion ELISA [22] using a proprietary aggregate-binding ligand. We next compared data generated by the newly-developed MSD assays with these existing assay technologies. For comparison to the TR-FRET assays, experiments with recombinant HTT(1–97) were performed, including aggregation kinetics and analysis of HTT(1–97)-Q16 and Q46 before and after thrombin-digestion. Similar to the results from the MSD assays (Fig 2), a gradual signal increase over time was observed, with a signal plateau after approximately 8 h incubation (Fig 7A). The 4C9-Tb / 4C9-AF488 assay also detected uncleaved MBP-HTT(1–97)-Q46 to some extent, and showed a signal response at the earliest time points (Fig 7A). These data suggest that this assay can already detect smaller HTT oligomers. When comparing detection of MBP-tagged and cleaved HTT(1–97)-Q16 and HTT(1–97)-Q46, the MW8-Tb / MW8-d2 TR-FRET assay showed preferential detection of aggregated HTT(1–97)-Q46 (Fig 7B). The 4C9-Tb / 4C9-AF488 assay also showed a strong signal response for aggregated HTT(1–97)-Q46; however, a signal response was also observed for digested HTT(1–97)-Q16 (Fig 7C). These data suggest that the 4C9-Tb / 4C9-AF488 TR-FRET assay might also detect oligomers or smaller aggregate species formed by thrombin-digested HTT(1–97)-Q16 [37], which is in accordance with results observed for the aggregation kinetics (Fig 7A). Overall, the limits of detection of 38 nM for the MW8-Tb / MW8-d2 and of 18 nM for the 4C9-Tb / 4C9-AF488 assays were more than 1000-fold higher than those determined for the MSD aggregate assays (Table 1).

**Fig 8:**
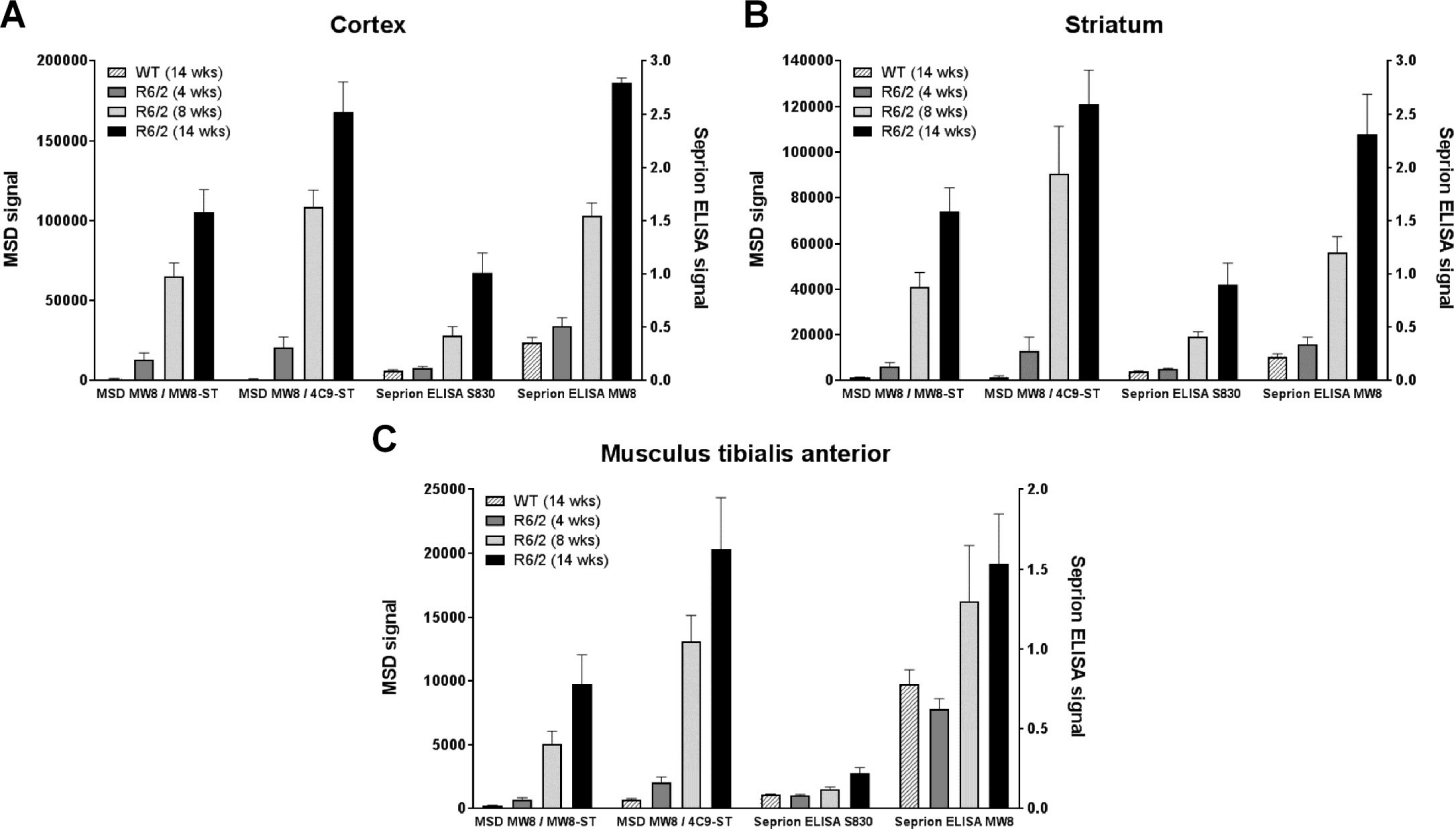
Comparison of MSD assays and Seprion ELISA. Homogenates of (A) cortex, (B) striatum, and (C) musculus tibialis anterior tissues of an aged series of R6/2 mice were analyzed at 1 mg/mL (cortex, TA) or 0.75 mg/mL (striatum) in the MW8 / 4C9-ST and MW8 / MW8-ST MSD assays, or using 15 μL of a 2.5% (w/v) ribolysate in the Seprion ELISA assay utilizing antibodies S830 or MW8. The background signal in the Seprion ELISA samples is generated by the substrate (see WT samples). Error bars represent standard deviations of four (WT) or eight (R6/2) biological replicates.

For comparison of the HTT aggregate MSD assays to the Seprion ELISA, the cortex, two striata, and two musculus tibialis anterior were collected from R6/2 and wild type mice at 4, 8, and 14 weeks of age. Half a cortex, one striatum and one TA was used from each mouse in the MSD aggregation and Seprion ELISA assays according to the standard protocols for each technique. As previously described, the amount of HTT aggregates in the brain increased with age (Fig 4D and 4G; [22]). Both MSD and Seprion ELISA assays showed comparable results for the cortex and striatum samples, with a similar age-dependent signal increase (Fig 8A and 8B). While the age-effect was also confirmed in the TA samples for both assay formats, detection of HTT aggregates in the TA samples appeared to be more sensitive in the MSD assays, where signals were detected as early as 4 weeks of age (Fig 8C). In summary, the newly developed assays described here show comparable results to already published HTT aggregate-selective assays, displaying equal or improved assay performance.

## Discussion

The evaluation of current HTT-lowering approaches for treatment of HD requires the availability of assays that are capable of measuring HTT levels in relevant HD model or patient samples. Multiple assay technologies are available to measure different monomeric HTT species [16–18, 20]. For example, MSD assays have been described for detection of mouse HTT, human HTT or mutant HTT [19]. Besides the presence of the pathogenic monomeric mHTT protein, aggregation of HTT protein is another hallmark of HD. However, only very few assays have been described that specifically target HTT aggregates [22, 23]. To complement the available set of MSD assays for detection of different HTT species, we developed two assays that preferentially detect aggregated HTT. The described MW8 / 4C9-ST and MW8 / MW8-ST MSD assays were evaluated using both recombinant HTT proteins and different tissue samples from HD mouse models. Each of the performed tests showed a distinct detection pattern in comparison to MSD monomer assays and confirmed that the two novel assays are preferentially detecting HTT in an aggregated form. Both assays show similar overall performance, with the MW8 / 4C9-ST assay being slightly more sensitive. When set against existing HTT aggregate assays, our assays showed comparable results with equal or improved assay performance. The novel MSD assays are clearly more sensitive in the detection of HTT aggregates in comparison to existing TR-FRET assays with more than 1000-fold lower LLODs. The Seprion ELISA is carried out in 96-well format. The described MSD assays are run in 384-well format and therefore allow for a higher throughput. Seprion ligands have been described to also detect non-HTT aggregates like prions and amyloid-β [42, 43]. Our data suggest that the two novel MSD assays described here do not detect other protein oligomeric or aggregated species, such as Aβ fibrils (S5 Fig) for which no signals were detected. Lastly, for the analysis of musculus tibialis anterior samples the MSD assays showed improved sensitivities (Fig 8C).

Both assays are based on the MW8 antibody, which has previously been shown to recognize HTT aggregates in IHC applications [24, 25]. EM48, a mouse monoclonal antibody raised against a GST fusion protein including the first 256 amino acids of human HTT, was also shown to label HTT aggregates in IHC studies [13, 44]. The EM48 antibody (MAB5374; Millipore) was also evaluated in the course of this study. Data generated for the analysis of recombinant HTT proteins with antibody combinations including EM48 also suggested potential aggregate-binding. However, assay signals were strongly suppressed by a mouse brain tissue matrix, which lead to signal intensities for aggregated HTT in mouse model tissues only minimally above or below the LLOD. Therefore, the EM48 antibody was not used for further evaluation. The epitope sequence for the MW8 antibody is not only present on aggregates, but also on monomeric HTT. It has been shown that MW8 is a neo-epitope antibody for the C-terminus of exon 1 HTT, that it does not detect this sequence when present in a longer HTT fragment or the full-length protein, and that its’ recognition of this epitope is dependent on the presence of the C-terminal proline [45]. Whilst the MW8 / MW8-ST assay might be expected to detect oligomers / aggregates, it is not clear as to why the MW8 / 4C9-ST combination is specific to HTT aggregates, given that MW8 can detect soluble exon 1 HTT [40, 45].

One aspect of the described assays that has not yet been addressed is which types of aggregates are being detected. In general, the term aggregate is a term that encompasses oligomers, protofibrils, fibrils and inclusions. Considering the data for the aggregation kinetics of HTT(1–97)-Q46 (Fig 2), and the elution profiles for analysis of R6/2 and zQ175 mouse whole brain lysates after SEC (Fig 5), both the MW8 / 4C9-ST and the MW8 / MW8-ST assay seem to a wide range of aggregate species. Comparison of these elution profiles to elution peaks obtained for a reference protein calibration mix indicated that the molecular weight of molecules eluting at 8 mL is larger than 700 kDa and most probably in the MDa range (molecules detected at 8 mL eluted earlier than a thyroglobulin calibration standard with a molecular weight of 669 kDa).

The principle of TR-FRET is a detectable energy transfer between two labeled antibodies bound in close proximity. This means that the applied TR-FRET assays can potentially already detect HTT dimers and smaller oligomers. In particular, the 4C9-Tb / 4C9-AF488 assay seemed to detect smaller HTT oligomers or aggregates, as seen by the signal increase at the very early time points of aggregation (Fig 7A) and a significant signal for the detection of thrombin-digested HTT(1–97)-Q16 (Fig 7C). These potentially smaller species detected by the 4C9-Tb / 4C9-AF488 TR-FRET assays are not identified in our MSD aggregate assays, which means that a certain aggregate size is required for detection. To determine and characterize the type of aggregates detected by our assays, the *in vitro* generated HTT(1–97)-Q46 aggregates could be analyzed by electron microscopy (EM) or atomic force microscopy (AFM).

Tissue homogenates of R6/2, zQ175, and BACHD mouse models were analyzed during the course of this study. These HD model animals differ in the expressed mHTT species and Q-length. R6/2 animals are transgenic for the 5’ region of the human *HTT* gene that contains exon 1 with approximate Q-lengths of 120 [33] in the colony maintained at the Jackson Laboratory, and 210 in the colony maintained by the Bates Lab. zQ175 mice produce a chimeric full-length HTT in which exon 1 of mouse HTT is replaced by the human sequence with an average Q-length of around 198 [35]. zQ175 mice also express exon 1 HTT encoded by a small polyadenylated mRNA that is generated through incomplete splicing [40]. Our size-exclusion chromatography data revealed a peak at 16 mL of elution consistent with the presence of the exon 1 HTT monomer. BACHD mice express a human full-length HTT with Q-length of 97 [26]. Analysis of the age series for each of these three mouse models in our aggregate assays suggests that formation of aggregates is dependent on overall construct length and polyQ-length. The highest signals were detected in the R6/2 mice (Fig 4D and 4G), as exon 1 HTT aggregates very readily. Aggregate-levels in the oldest animals were approximately 5-10-fold lower in hetQ175 animals in comparison to R6/2 mice (Fig 4E and 4H), which may be consistent with the lower level of exon 1 HTT present in these knock-in mice. The lowest signals were measured in the BACHD mice. Here, considering the applied total lysate concentrations, signals for the oldest animals were around 100-fold lower than for the hetQ175 animals in the MW8 / 4C9-ST assay (Fig 4F). In the MW8 / MW8-ST assays, signals were below the limit of detection for all samples (Fig 4I). One reason for the differences in aggregate load between hetQ175 and BACHD mice could be the significant longer Q-length in the hetQ175 animals. For all three animal models, the observed increase in aggregate levels was stronger than the concomitant decrease of mHTT levels (Fig 4A–4C). This could be due to the intracellular generation of new mHTT protein to compensate for the mHTT molecules that had been incorporated into aggregates and to retain a constant level of cellular monomeric HTT.

Following the analysis of tissue samples from HD mouse models, we also attempted to detect HTT aggregates in cortical samples of human HD patients. Patients were heterozygous with respect to expanded CAG repeats and the average Q-length was 45. However, no signals above the LLOD could be detected in any of the samples for either the MW8 / 4C9-ST or the MW8 / MW8-ST assay. It has been recently shown that the amount of nuclear inclusions in neurons of the prefrontal cortex is dramatically higher in HD mouse models (i.e. R6/2 and zQ175) than in HD patient samples. While 40–50% of neurons in mouse models contained nuclear mHTT inclusions, only 0.3% of neurons in human samples showed inclusions [46]. Therefore, the relative aggregate load in the tested human samples was most certainly lower than for the BACHD mouse homogenates. This means that the sensitivities of the presented assays are not high enough to detect the very low abundant HTT aggregates in human patient samples. We therefore would like to emphasize that we see the application of these assays in the analysis of preclinical models of HD rather than for detection of HTT aggregates in human samples. Detection of human aggregates in the described MSD assays could possibly be achieved by an enrichment step before analysis. Alternatively, the assays could be transferred to an antibody-based method including an enrichment step, such as the Erenna Singulex technology.

In summary, we showed that the two newly developed assays preferentially detect HTT in an aggregated form. These assays expand the capabilities of the available suite of MSD assays for the identification and quantification of the different HTT species present in preclinical models of HD. We recommend the presented assays for aggregated HTT to be used in parallel with the soluble HTT assays to best assess the HTT protein population in particular biosamples for HD model characterization and HTT lowering studies.

## Supporting information

S1 Text Supporting materials and methods. (PDF)

S1 Fig Western blot confirming aggregation of HTT(1–97)-Q46. Incubation of MBP-tagged HTT(1–97)-Q16 or Q46 for 24 h at 37°C in absence or presence of thrombin protease. 3 μg per sample were loaded onto the SDS-PAGE gel. After blotting, membranes were probed with EM48 antibody and an alkaline phosphatase-conjugated secondary antibody. Western blotting confirmed almost complete cleavage for both HTT(1–97) constructs, whereas high molecular weight aggregates could only be observed for HTT(1–97)-Q46. Molecular weights: MBP 43 kDa; MBP-HTT(1–97)-Q16: 54 kDa; MBP-HTT(1–97)-Q46: 58 kDa; thrombin-digested HTT(1–97)-Q16: 11 kDa; non-aggregated thrombin-digested HTT(1–97)-Q46: 15 kDa. M: molecular weight marker. * This band is most probably an impurity from the purification process. Please note: due to the unusual amino acid composition (high number of glutamines), all bands are detected at a slight discrepancy to the molecular weight standard.
(TIF)

S2 Fig Characterization of 2B7 / 4C9-ST and 2B7 / MW1-ST assays. Human N-terminal HTT fragment 1–573 (HTT NF573)-Q23 and polyQ-expanded Q73 were tested at different concentrations in the 2B7 / 4C9-ST and (B) 2B7 / MW1-ST assays. (A) While assay 2B7 / MW1-ST is preferentially detecting mHTT, (B) assay 2B7 / 4C9-ST detects total soluble HTT independent of Q-length. Error bars represent standard deviations for three independent measurements. In some cases, error bars are too small to be visible.
(TIF)

S3 Fig Immunoprecipitation of aggregated HTT(1–97)-Q46 with MW8. Monomeric and thrombin-digested MBP-HTT(1–97)-Q46 were immunoprecipitated using the MW8 antibody. 0.2 μg of protein per lane were used as input control (lanes 1 and 2), while 2 μg of protein were loaded for the immunoprecipitation with MW8 (lanes 3 and 4) and for the “beads only” control (lanes 5 and 6). 4C9 was used as detection antibody. Detection of HTT aggregates by MW8 was confirmed (lane 4), but also the monomeric HTT species MBP-HTT(1–97)-Q46 (lane 3) and thrombin-digested non-aggregated HTT(1–97)-Q46 (lane 4) were immunoprecipitated by MW8 to some extent. Interpretation of results based on the cleaved, but non-aggregated HTT(1–97)-Q46 bands (exon1-Q46) is not possible without doubt, as HTT(1–97)-Q46 also binds unspecifically to the beads (lane 6). Considering that 10 times more material was used for lanes 3 and 4 than for lanes 1 and 2, data suggests that almost all HTT aggregates present in the input were pulled down by MW8, whereas only approximately 1% of MBP-HTT(1–97)-Q46 (MBP-exon1-Q46) from the input were pulled down. Therefore, these data indicate that MW8 is preferentially detecting aggregated HTT. Molecular weights: MBP-HTT(1–97)-Q46: 58 kDa; non-aggregated thrombin-digested HTT(1–97)-Q46: 15 kDa. M: molecular weight marker. * This band is most probably an impurity from the purification process. Please note: due to the unusual amino acid composition (high number of glutamines), all bands are detected at a slight discrepancy to the molecular weight standard.
(TIF)

S4 Fig Filter retardation assay for measuring aggregation kinetics of HTT(1–97)-Q46. Aggregation of HTT(1–97)-Q46 was initiated by addition of thrombin protease, followed by incubation for 24 h at 37°C. Samples were taken at the given time points and analyzed in a filter retardation assay using the MW8 antibody as primary detection antibody. (A) The stained membrane showed a time-dependent MW8 signal increase after initiation of aggregation. Non-digested monomeric MBP-HTT(1–97)-Q46 was not detected by MW8. (B) Quantification of MW8 signals on the membrane confirmed the time-dependent signal increase with a plateau being observed after 8 h of incubation. This result is in accordance with the MW8 / 4C9-ST and MW8 / MW8-ST MSD assay data. Error bars represent standard deviation for technical duplicates. a.u. artificial units.
(TIF)

S5 Fig Assay selectivity against amyloid-β fibrils and polyQ46 aggregates. (A) *In vitro* generation of Aβ 42 fibrils was confirmed by analysis of Aβ samples at different concentrations in a Thioflavin T assay. The same fibrils were analyzed in the (B) MW8 / 4C9-ST and (C) MW8 / MW8-ST MSD assays. Aggregated HTT(1–97)-Q46 was included as control. While both assays showed a dose-dependent detection of aggregated HTT(1–97)-Q46, no signals were observed for Aβ fibrils. D) *In vitro* generation of polyQ46 aggregates was confirmed by analysis of GST-tagged and thrombin-cleaved polyQ46 at different concentrations in a Thioflavin T assay. The same samples were analyzed in the (E) MW8 / 4C9-ST and (F) MW8 / MW8-ST MSD assays. Aggregated HTT(1–97)-Q46 was included as control. While both assays showed a dose-dependent detection of aggregated HTT(1–97)-Q46, no signals were observed for polyQ46 aggregates. Error bars represent standard deviations for three independent measurements (A-C) or three replicate measurements (D-F). In some cases, error bars are too small to be visible.
(TIF)

S6 Fig Spike recovery in tissue matrix. A standard curve of HTT(1–97)-Q46 aggregated for 24 h at 37°C was analyzed in buffer or spiked with 1 mg/mL of a wildtype mouse whole brain lysate as background matrix in the (A) MW8 / 4C9-ST and (B) MW8 / MW8-ST assays. None of the assays were significantly affected by the tissue matrix with both buffer and matrix curves showing a nearly identical signal response. Error bars represent standard deviations for three independent measurements. In some cases, error bars are too small to be visible.
(TIF)

S7 Fig Analysis of endogenous mouse HTT levels in an aged series of HD mouse models. Whole brain lysates of an aged series of (A) R6/2, (B) hetQ175, and (C) BACHD mice were analyzed at 0.5 mg/mL (R6/2 and hetQ175) or 1 mg/mL (BACHD) in the pAb147 / 2166 / ST assay for mouse HTT. While endogenous mouse HTT levels were independent of age and genotype for R6/2 and BACHD mice, hetQ175 mice showed approximately half the amount of mouse HTT in comparison to respective wild type animals, as the mouse specific assays does not recognize the mutant human/mouse chimeric allele. Background: signal generated by sample buffer. Error bars represent standard deviations of at least three biological replicates. Samples were compared based on a one-way ANOVA statistical analysis: * p < 0.05, **** p < 0.0001, n.s. not significant.
(TIF)

S8 Fig Calibration of the Superose 6 SEC column using recombinant HTT standards. 500 μL of (A) MBP-tagged monomeric HTT(1–97)-Q46, (B) thrombin-digested, aggregated HTT(1–97)-Q46, and full-length HTT (C) HTT-Q17 and (D) Q46 were run on a Superose 6 SEC column at the given concentrations. Here, proteins were separated by size, with larger proteins found in earlier elution fractions. Elution fractions were analyzed in the 2B7 / MW1-ST and/or 2B7 / 4C9-ST assays for human (m)HTT, and the aggregate-selective assays MW8 / 4C9-ST and MW8 / MW8-ST. Aggregated HTT(1–97)-Q46 eluted first at ~ 8 mL, full-length HTT-Q17 and Q46 (Mw: 347 and 351 kDa) showed the maximum elution at ~ 11–12 mL, MBP-HTT(1–97)-Q46 (Mw: 58 kDa) eluted last at ~ 16 mL. Curve shapes were smoothed. Confirmation of detected HTT species by Western blotting of individual elution fractions failed as samples were too dilute after SEC.
(TIF)

S9 Fig Intrastriatal adenoviral-delivery of ZFP targeting mutant HTT resulted in reduction of HTT aggregates in zQ175 mice. (A) and (B) Schematic representation of ZFP viral vectors: (A) p_hsyn1_-HTT-ZFP30640, (B) ZFP control constructs lacking DNA binding domain, p_hsyn1_-HTT-ZFP-ΔDBD; ITR: inverted terminal repeat, p_hSyn1_: human synapsin1 promoter, ZFP: zinc finger protein, WPRE: woodchuck hepatitis virus posttranscriptional regulatory element, A: polyA sequence, ΔDBD: sequence lacking DNA binding domain. (C and D) Representative images showing mEM48 immunostaining in striatal tissue from heterozygous zQ175 mice injected with (C) AAV-SWB-ZFP30640 and (D) AAV-SWB-ZFP-ΔDBD at 2 months of age and analyzed 4 months post-injection. A strongly reduced HTT inclusions load (mEM48-ir) was observed for AAV-transduced (FLAG+) neurons (NeuN-ir) in ZFP30640-treated animals in comparison with the ΔDBD control. Quantitative analysis of the images shows marked reduction of perinuclear HTT inclusions (E) and nuclear mEM48 granularity (texture) in ZPF30640-treated animals (F).
(TIF)
