# Supplementary material for: Meso scale discovery-based assays for the detection of aggregated huntingtin
Source: PLoS One. 2019 Mar 26;14(3):e0213521. doi: 10.1371/journal.pone.0213521 (PMC6435127; doi:10.1371/journal.pone.0213521)
Supplement: S1 Text — (PDF) [file pone.0213521.s001.pdf]

Supporting information to:

Meso Scale Discovery-Based Assays for the Detection of Aggregated Huntingtin

Wolfgang Reindl[1], Barbara Baldo[1], Jana Schulz[1], Isabell Janack[1], Ilka Lindner[1], Markus Kleinschmidt[1], Yalda Sedaghat[1], Christina Thiede[1], Karsten Tillack[1], Christina Schmidt[1], Isabell Cardaun[1], Tom Schwagarus[1], Frank Herrmann[1], Madlen Hotze[1], Georgina F. Osborne[2], Simone Herrmann[1], Andreas Weiss[1], Celina Zerbinatti[1], Gillian P. Bates[2], Jonathan Bard[3], Ignacio Munoz-Sanjuan[3], Douglas Macdonald[3]*

[1] Evotec AG, Hamburg, Germany

[2] Dept. Neurodegenerative Disease, Huntington's Disease Centre and Dementia Research Institute, Institute of Neurology, University College London, London, UK

[3] CHDI Management/CHDI Foundation, Los Angeles, CA, USA

* Corresponding author

E-mail: Douglas.Macdonald@chdifoundation.org

Supporting materials and methods

Recombinant huntingtin protein HTT(1-97)-Q46

HTT(1-97)-Q46 sequence after thrombin cleavage:

GGMATLEKLMKAFESLKSFQQQQQQQQQQQQQQQQQQQQQQQQQQQQQQQQQQ

QQQQQQQQQQQQPPPPPPPPPPPQLPQPPPQAQPLLPQPQPPPPPPPPPPGPAVAE

EPLHRPKKELSATHHHHHH

Western blot analysis of HTT proteins

3 μg each of MBP-tagged or thrombin digested HTT(1-97)-Q16 and Q46 were mixed with 4x Laemmli loading buffer and incubated for 5 min at 99°C. Electrophoresis was run for 24 min at 100 mA in pre-cast NuPAGE Novex 4-12% bis-tris protein gels (ThermoFisher Scientific) with MOPS running buffer (50 mM MOPS, 50 mM Tris, 0.1% SDS, 1 mM EDTA, pH 7.7). Subsequently, proteins were transferred to a PVDF membrane for 10 min at 20 V using the iBlot 2 Dry Blotting System (ThermoFisher Scientific). Membranes were probed with antibodies using the iBind system (ThermoFisher Scientific). As primary antibody EM48 (1:200) was used. The mouse monoclonal antibody EM48 (MAB5374), raised against a GST fusion protein of the first 256 amino acids from human HTT with an in-frame deletion of the poly-glutamine and poly-proline stretches [1], was obtained from Millipore. The secondary antibody was an alkaline phosphatase (AP)-conjugated anti-mouse IgG antibody (1:1,500; Promega). For visualization of protein bands, the membrane was treated with NBT/BCIP solution (Sigma).

Immunoprecipitation of HTT proteins

The MW8 antibody was diluted in PBS-T to obtain 2 μg antibody in 200 μL final volume. 30 μL of Dynabeads per sample were transferred to a 1.5 mL Eppendorf tube, placed on a magnet and the supernatant was removed. The beads were then washed 3x with 500 μL PBS-T, and after the last washing step resuspended in the 200 μL of diluted MW8. The antibody-beads mix was incubated for 30 min at RT on a rotator. For the control samples, the beads were incubated for 30 min with PBS-T only. The tubes were then placed on a magnet and washed 3x with 500 μL PBS-T. 50 μg each of MBP-tagged or thrombin digested HTT(1-97)-Q46 diluted in 500 μL of lysis buffer were added to the antibody-beads mix and incubated o/n at 4°C on a rotator. The tubes were then placed on a magnet and washed 4x with 500 μL PBS-T. After the last washing step the supernatant was carefully removed, 30 μL of loading buffer were added to each tube, and the samples were incubated for 5 min at 95°C. 2 μg of each eluate were loaded on a SDS-PAGE. 0.2 μg of MBP-tagged or thrombin digested HTT(1-97)-Q46 were loaded as input control. Electrophoresis was run for 60 min at 150 V in pre-cast NuPAGE Novex 4-12% bis-tris protein gels (ThermoFisher Scientific) with NuPAGE MES SDS running buffer (ThermoFisher Scientific). Subsequently, proteins were transferred using a wet blotting system to a PVDF membrane for 90 min at 200 mA. The membrane was blocked with 10 mL Odyssey Blocking Buffer (Li-COR Biosciences) for 1 h at RT. The membrane was incubated with 4C9 (1:1,000) as primary antibody o/n at 4°C. After 3 washes with TBS-T, the membrane was incubated with AlexaFluor 790 Goat Anti-Mouse IgG (H+L) (1:10,000; ThermoFisher Scientific) as secondary antibody. Visualization of protein bands was performed using the LI-COR scanner (LI-COR Biosciences).

Filter retardation assay

Exon 1-Q46 aggregates were prepared as described above by mixing 10 μM MBP-tagged HTT(1-97)-Q46 with 150 μg/mL bovine thrombin protease (Sigma) and 2 mM $CaCl_2$ in a total volume of 200 μL 50 mM Tris (pH 8.0), 150 mM NaCl. Samples were incubated for 0-24 h at 37°C and directly frozen on dry ice. Equal protein concentrations (3 μg/well) of soluble or aggregated HTT(1-97)-Q46 were vacuum-filtered in doublets through a pre-equilibrated (PBS) and pre-blocked (PBS containing 1% Pluronic F-127 and 3% BSA) PVDF membrane (0.2 μm; GE Healthcare / Whatman) using the 96-well Bio-Dot Apparatus (Bio-Rad). During suction, each well was washed three times with 200 μL of PBS. The membrane was blocked in TBS (100 mM Tris-HCl and 150 mM NaCl, pH 7.4) containing 5% nonfat dried milk for 1 h at room temperature and was subsequently incubated for 2 h with primary anti-HTT antibody MW8 (Developmental Studies Hybridoma Bank) diluted 1:1,000 in TBS-T containing 5% nonfat dried milk. The membrane was incubated with a second HRP-conjugated antibody for 1 h, processed for visualization using an enhanced chemiluminescence system (GE Healthcare), and exposed to medical X-ray film (Hyperfilm ECL, GE Healthcare) to obtain fluorographic images. Quantification was performed by densitometry with ImageJ.

Generation and analysis of amyloid-β and polyQ46 aggregates

Amyloid-β (Aβ) fibrils were generated by incubation of 10 μM human Aβ peptide 1-42 (Tocris Bioscience) in 50 mM Tris pH 8.0, 150 mM NaCl for 18.5 h at 37°C. The aggregate solution was aliquoted and stored at -20°C until further use. For generation of polyQ46 aggregates, 33 μM GST-polyQ46 were mixed with 150 μg/mL bovine thrombin protease

(Sigma) and 2 mM $CaCl_2$ in 50mM Tris pH 8.0, 150 mM NaCl and incubated for 16 h at 37°C. Thrombin cleavage of the GST-tag induces aggregation of polyQ46. After incubation samples were centrifuged for 5 min at 13,000 rpm, the supernatant was discarded and the pellet resuspended in 50mM Tris pH 8.0, 150 mM NaCl. The protein was then stored at -80°C until further use. The Thioflavin T assay [2] was used to confirm Aβ and polyQ46 aggregate formation. Aggregated and non-aggregated Aβ and polyQ46 were diluted in 20% tissue lysis buffer / 80% MSD blocking buffer and mixed with 5 μM Thioflavin T (Sigma). Fluorescence was measured (ex: 440 nm / em: 482 nm) after incubation for 15 min at RT. The Aβ and polyQ46 aggregates were also analyzed in the MSD assays.

ZFP expression in HD mouse models

Animal studies

Live zQ175 C57B/L6J knock-in mice and wildtype littermates were obtained from the Jackson Laboratory (Bar Harbor, USA). Animals were housed in Eurostandard Type II long cages and given access to food and water *ad libitum*. Environmental conditions were maintained at a temperature of 21 ± 1°C, humidity of 55 ± 1 0% and a 12:12 h light:dark cycle, with lights on at 7 am and off at 7 pm. Animals were checked daily for health status during housing and study duration. All animal work was carried out in accordance with the regulations of the German animal welfare act and the EU legislation (EU directive 2010/63/EU). The study protocol was approved by the local Ethics committee of the Authority for Health and Consumer Protection of the city and state of Hamburg ("*Behörde*

für Gesundheit und Verbraucherschutz” BGV, Hamburg) under the file number #V11307/591 00.33.

AAV vector construction and production

For expression of ZFPs, plasmids were modified from the AAV vector pAAV-6P-SWB [3]. ZFP30640 (FLAG-tagged) was cloned after the human synapsin 1 promoter (p_{hSyn1}) to generate pAAV-6P-SWB-ZFP-30640. In addition, an inactive ZFP control construct was generated by deleting the ZFP DNA binding domain from ZFP33074 (pAAV-6P-SWB-ZFP-ΔDBD). Pseudotyped recombinant (r)AAV2/1+2 particles were produced and purified as previously described [4, 5]. In brief, HEK293 cells were co-transfected with AAV vector carrying the transcription units of interest and plasmids containing rep and cap genes (pDP1rs and pDP2rs, Plasmid Factory) in equimolar ratios by polyethylenimine-mediated plasmid transfection. Cells were lysed 48 hours after transfection by three freeze-thaw cycles, and cellular debris were removed by centrifugation. The supernatant containing the viral particles was treated with benzonase, and subjected to iodixanol density centrifugation (S6, S7) at 60,000 rpm. Iodixanol was removed and viral particles were concentrated in PBS 300 MK (300 mM NaCl, 1 mM $MgCl_2$, 2.5 mM KCl) by filter centrifugation. The remaining rAAV solution was filtered through a Millex GV 0.22 μm pore size. Sterile rAAV particles were stored at 4°C and diluted 1:1 with sterile PB buffer to obtain PBS MK (150 mM NaCl, 0.5 mM $MgCl_2$, 1.25 mM KCl) prior to *in vivo* application. AAV titers were determined using qPCR.

Histology and immunohistochemistry

Mice were euthanized 4 months post-injection at 6 months of age. For immunohistochemistry, mice were sacrificed by transcardial perfusion of anesthetized animals with 30 mL of ice-cold PBS and 50 mL of 4% paraformaldehyde using a peristaltic pump. Brain samples were removed from the skull and processed for immunohistochemistry as previously described [5].

Image acquisition and automated image analysis

Image acquisition and analysis were performed as previously described [5]. In brief, automated image acquisition was conducted using the Opera High Content Screening system and Opera software 2.0.1 (PerkinElmer Inc.) with a 40x water immersion objective (Olympus, NA 1.15, pixel size: 0.32 μm). Image analysis scripts for characterization and quantification of mHTT inclusions were developed using Acapella Studio 3.1 (PerkinElmer Inc.) and the integrated Acapella batch analysis as part of the Columbus system.

References

1. Gutekunst CA, Li SH, Yi H, Mulroy JS, Kuemmerle S, Jones R, et al. Nuclear and neuropil aggregates in Huntington's disease: relationship to neuropathology. J Neurosci. 1999;19(7):2522-34. PubMed PMID: 10087066.
2. LeVine H, 3rd. Thioflavine T interaction with synthetic Alzheimer's disease beta-amyloid peptides: detection of amyloid aggregation in solution. Protein Sci. 1993;2(3):404-10. doi: 10.1002/pro.5560020312. PubMed PMID: 8453378; PubMed Central PMCID: PMCPMC2142377.
3. Minderer M, Liu W, Sumanovski LT, Kugler S, Helmchen F, Margolis DJ. Chronic imaging of cortical sensory map dynamics using a genetically encoded calcium indicator. J Physiol. 2012;590(1):99-107. doi: 10.1113/jphysiol.2011.219014. PubMed PMID: 22083602; PubMed Central PMCID: PMCPMC3300049.
4. Zolotukhin S, Byrne BJ, Mason E, Zolotukhin I, Potter M, Chesnut K, et al. Recombinant adeno-associated virus purification using novel methods improves infectious titer and yield. Gene Ther. 1999;6(6):973-85. doi: 10.1038/sj.gt.3300938. PubMed PMID: 10455399.
5. Carty N, Berson N, Tillack K, Thiede C, Scholz D, Kottig K, et al. Characterization of HTT inclusion size, location, and timing in the zQ175 mouse model of Huntington's disease: an in vivo high-content imaging study. PLoS One. 2015;10(4):e0123527. doi: 10.1371/journal.pone.0123527. PubMed PMID: 25859666; PubMed Central PMCID: PMCPMC4393127.
