# Supplementary figures and images for: Meso scale discovery-based assays for the detection of aggregated huntingtin
Source: PLoS One. 2019 Mar 26;14(3):e0213521. doi: 10.1371/journal.pone.0213521 (PMC6435127; doi:10.1371/journal.pone.0213521)

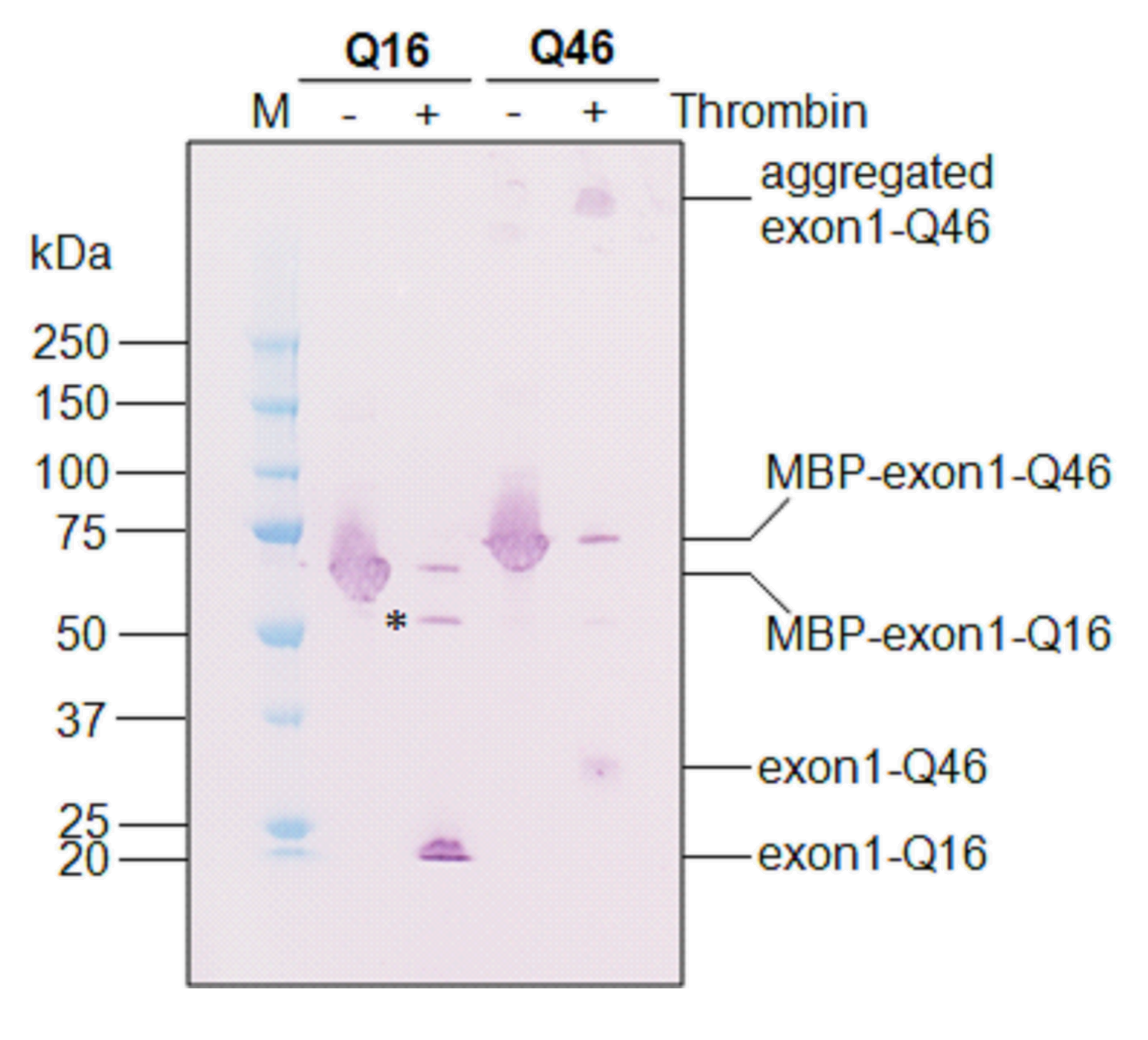

Supplement: S1 Fig — Incubation of MBP-tagged HTT(1–97)-Q16 or Q46 for 24 h at 37°C in absence or presence of thrombin protease. 3 μg per sample were loaded onto the SDS-PAGE gel. After blotting, membranes were probed with EM48 antibody and an alkaline phosphatase-conjugated secondary antibody. Western blotting confirmed almost complete cleavage for both HTT(1–97) constructs, whereas high molecular weight aggregates could only be observed for HTT(1–97)-Q46. Molecular weights: MBP 43 kDa; MBP-HTT(1–97)-Q16: 54 kDa; MBP-HTT(1–97)-Q46: 58 kDa; thrombin-digested HTT(1–97)-Q16: 11 kDa; non-aggregated thrombin-digested HTT(1–97)-Q46: 15 kDa. M: molecular weight marker. * This band is most probably an impurity from the purification process. Please note: due to the unusual amino acid composition (high number of glutamines), all bands are detected at a slight discrepancy to the molecular weight standard. (TIF) [file pone.0213521.s002.tif]

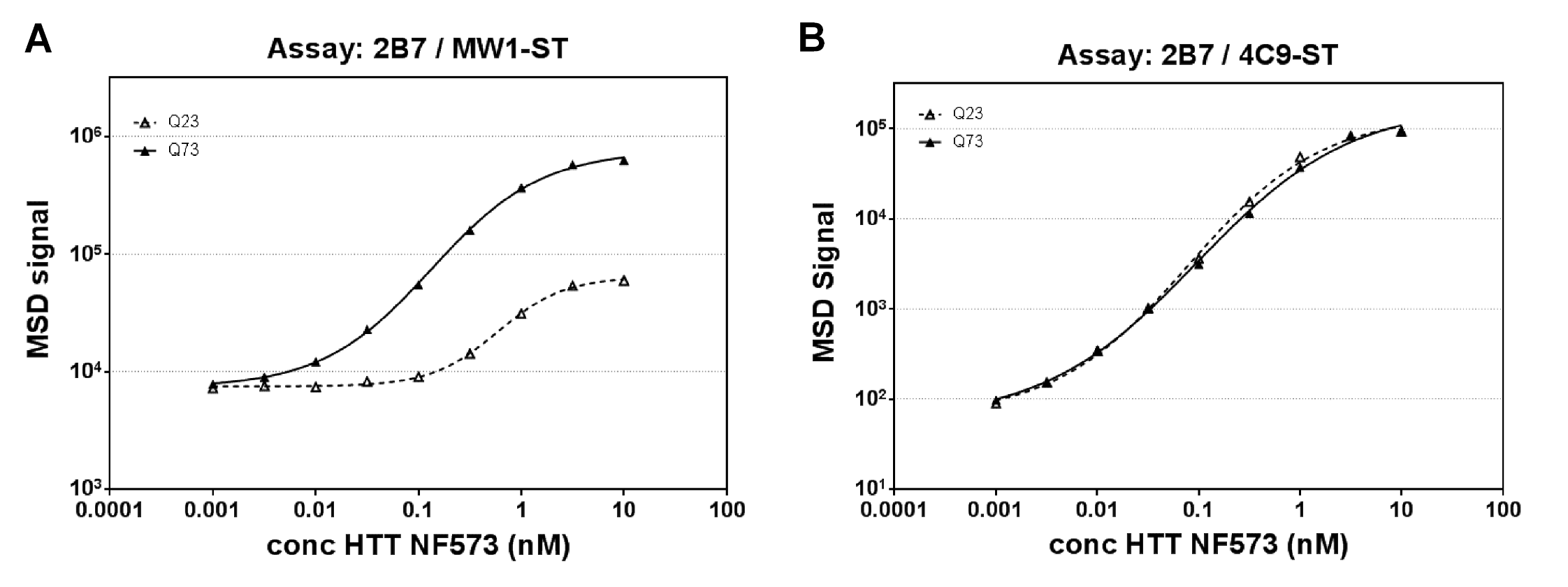

Supplement: S2 Fig — Human N-terminal HTT fragment 1–573 (HTT NF573)-Q23 and polyQ-expanded Q73 were tested at different concentrations in the 2B7 / 4C9-ST and (B) 2B7 / MW1-ST assays. (A) While assay 2B7 / MW1-ST is preferentially detecting mHTT, (B) assay 2B7 / 4C9-ST detects total soluble HTT independent of Q-length. Error bars represent standard deviations for three independent measurements. In some cases, error bars are too small to be visible. (TIF) [file pone.0213521.s003.tif]

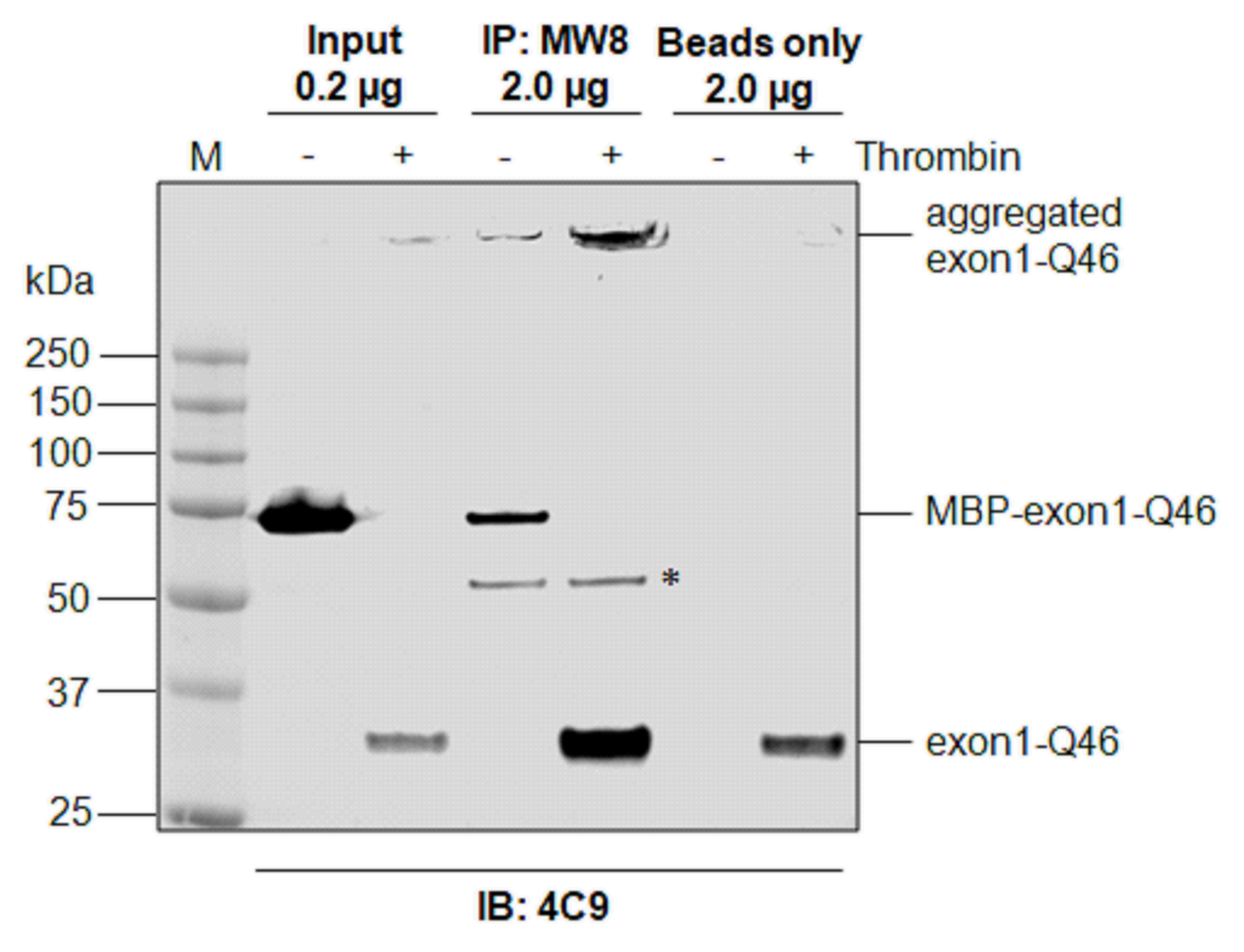

Supplement: S3 Fig — Monomeric and thrombin-digested MBP-HTT(1–97)-Q46 were immunoprecipitated using the MW8 antibody. 0.2 μg of protein per lane were used as input control (lanes 1 and 2), while 2 μg of protein were loaded for the immunoprecipitation with MW8 (lanes 3 and 4) and for the “beads only” control (lanes 5 and 6). 4C9 was used as detection antibody. Detection of HTT aggregates by MW8 was confirmed (lane 4), but also the monomeric HTT species MBP-HTT(1–97)-Q46 (lane 3) and thrombin-digested non-aggregated HTT(1–97)-Q46 (lane 4) were immunoprecipitated by MW8 to some extent. Interpretation of results based on the cleaved, but non-aggregated HTT(1–97)-Q46 bands (exon1-Q46) is not possible without doubt, as HTT(1–97)-Q46 also binds unspecifically to the beads (lane 6). Considering that 10 times more material was used for lanes 3 and 4 than for lanes 1 and 2, data suggests that almost all HTT aggregates present in the input were pulled down by MW8, whereas only approximately 1% of MBP-HTT(1–97)-Q46 (MBP-exon1-Q46) from the input were pulled down. Therefore, these data indicate that MW8 is preferentially detecting aggregated HTT. Molecular weights: MBP-HTT(1–97)-Q46: 58 kDa; non-aggregated thrombin-digested HTT(1–97)-Q46: 15 kDa. M: molecular weight marker. * This band is most probably an impurity from the purification process. Please note: due to the unusual amino acid composition (high number of glutamines), all bands are detected at a slight discrepancy to the molecular weight standard. (TIF) [file pone.0213521.s004.tif]

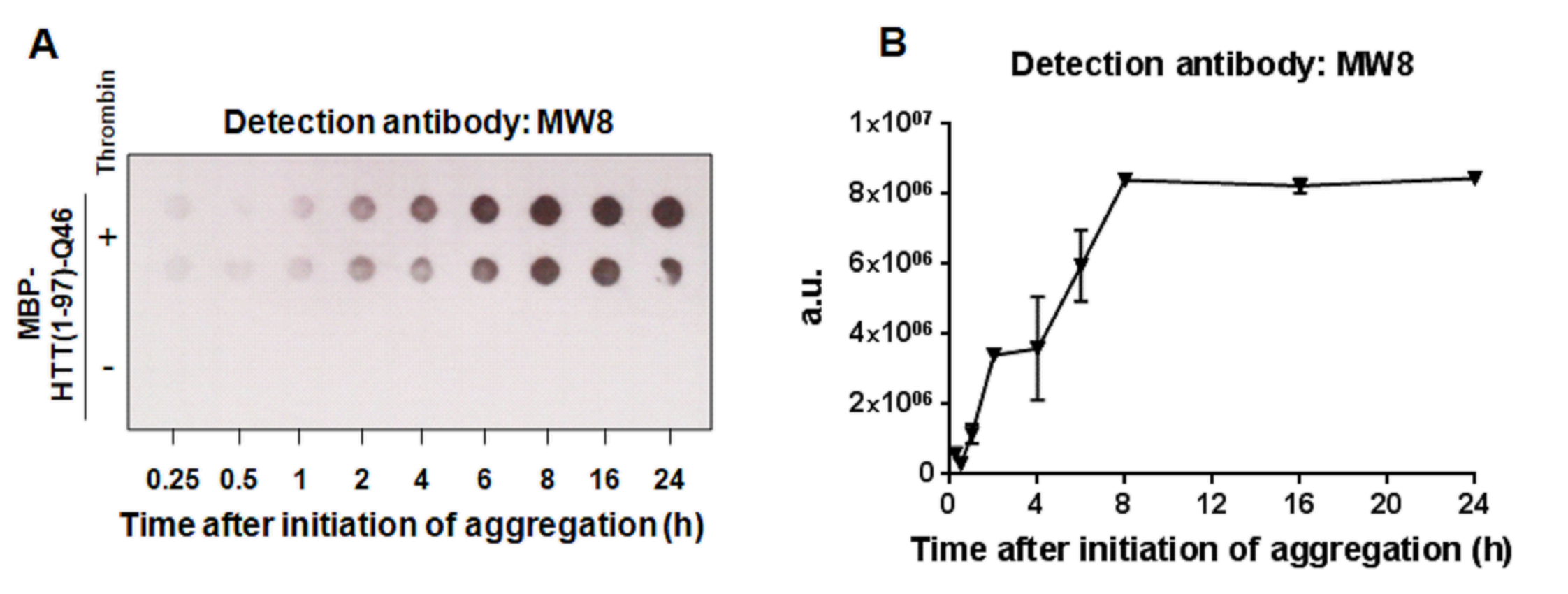

Supplement: S4 Fig — Aggregation of HTT(1–97)-Q46 was initiated by addition of thrombin protease, followed by incubation for 24 h at 37°C. Samples were taken at the given time points and analyzed in a filter retardation assay using the MW8 antibody as primary detection antibody. (A) The stained membrane showed a time-dependent MW8 signal increase after initiation of aggregation. Non-digested monomeric MBP-HTT(1–97)-Q46 was not detected by MW8. (B) Quantification of MW8 signals on the membrane confirmed the time-dependent signal increase with a plateau being observed after 8 h of incubation. This result is in accordance with the MW8 / 4C9-ST and MW8 / MW8-ST MSD assay data. Error bars represent standard deviation for technical duplicates. a.u. artificial units. (TIF) [file pone.0213521.s005.tif]

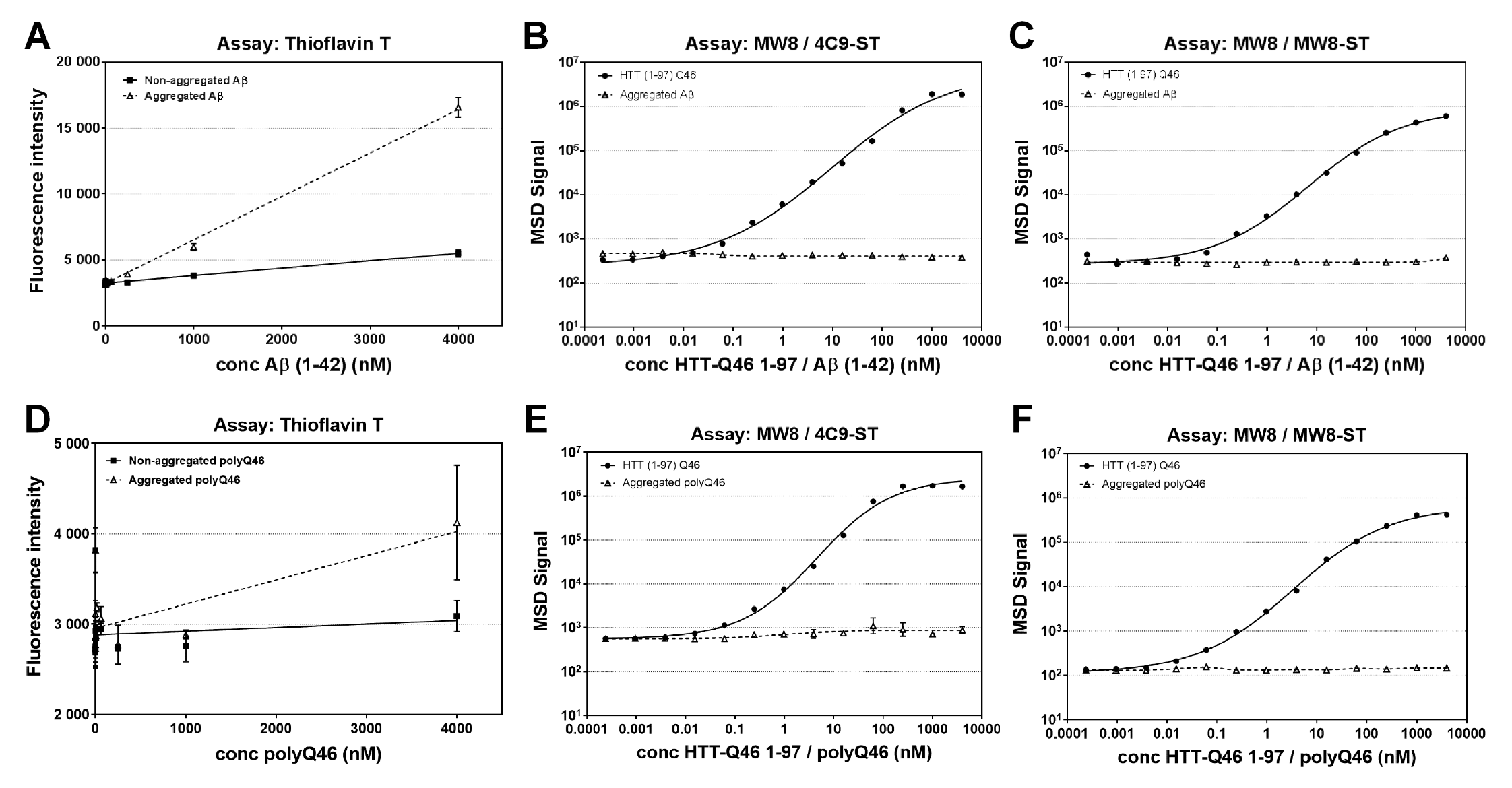

Supplement: S5 Fig — (A) In vitro generation of Aβ 42 fibrils was confirmed by analysis of Aβ samples at different concentrations in a Thioflavin T assay. The same fibrils were analyzed in the (B) MW8 / 4C9-ST and (C) MW8 / MW8-ST MSD assays. Aggregated HTT(1–97)-Q46 was included as control. While both assays showed a dose-dependent detection of aggregated HTT(1–97)-Q46, no signals were observed for Aβ fibrils. D) In vitro generation of polyQ46 aggregates was confirmed by analysis of GST-tagged and thrombin-cleaved polyQ46 at different concentrations in a Thioflavin T assay. The same samples were analyzed in the (E) MW8 / 4C9-ST and (F) MW8 / MW8-ST MSD assays. Aggregated HTT(1–97)-Q46 was included as control. While both assays showed a dose-dependent detection of aggregated HTT(1–97)-Q46, no signals were observed for polyQ46 aggregates. Error bars represent standard deviations for three independent measurements (A-C) or three replicate measurements (D-F). In some cases, error bars are too small to be visible. (TIF) [file pone.0213521.s006.tif]

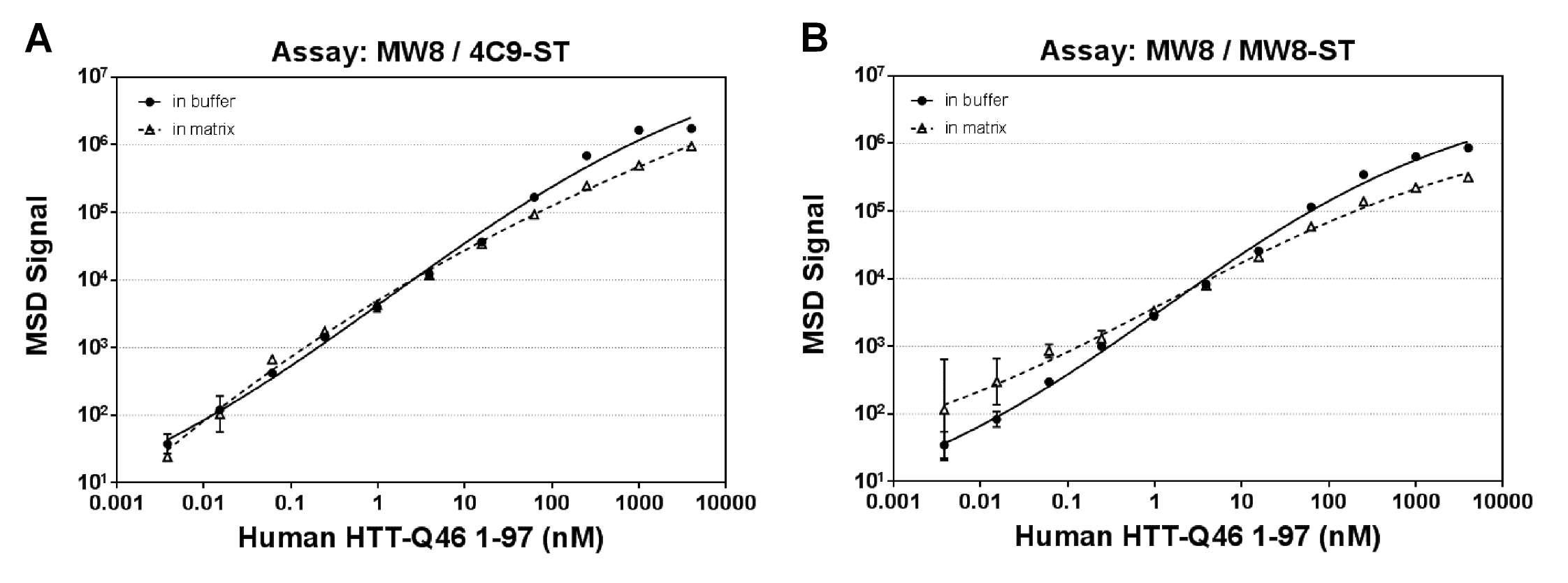

Supplement: S6 Fig — A standard curve of HTT(1–97)-Q46 aggregated for 24 h at 37°C was analyzed in buffer or spiked with 1 mg/mL of a wildtype mouse whole brain lysate as background matrix in the (A) MW8 / 4C9-ST and (B) MW8 / MW8-ST assays. None of the assays were significantly affected by the tissue matrix with both buffer and matrix curves showing a nearly identical signal response. Error bars represent standard deviations for three independent measurements. In some cases, error bars are too small to be visible. (TIF) [file pone.0213521.s007.tif]

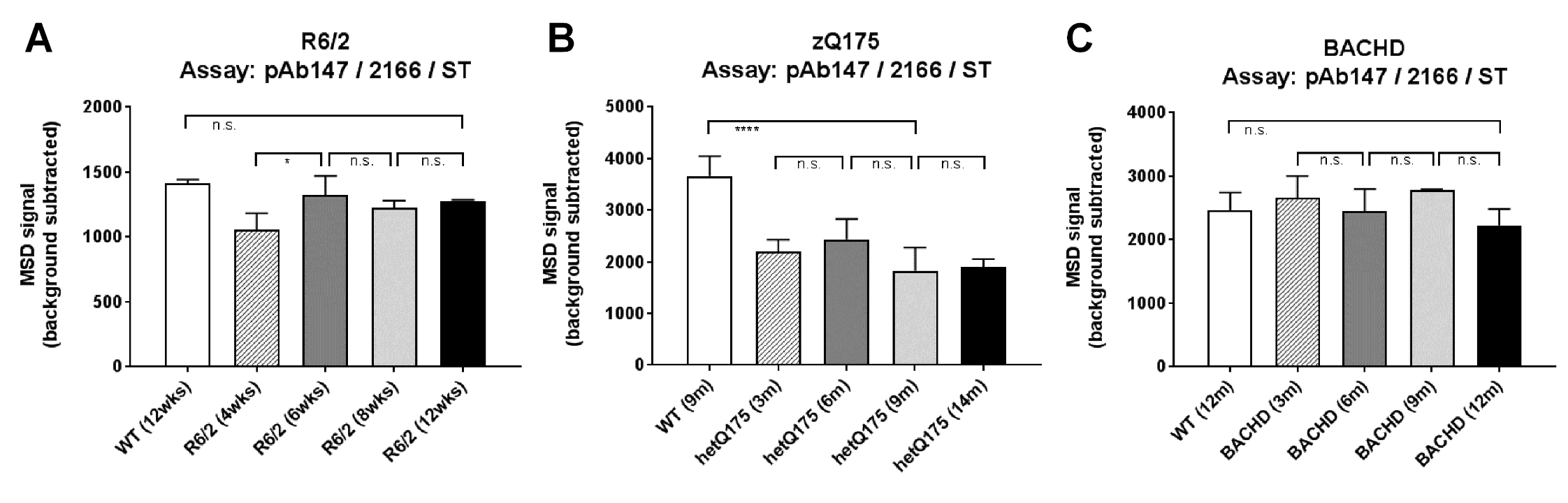

Supplement: S7 Fig — Whole brain lysates of an aged series of (A) R6/2, (B) hetQ175, and (C) BACHD mice were analyzed at 0.5 mg/mL (R6/2 and hetQ175) or 1 mg/mL (BACHD) in the pAb147 / 2166 / ST assay for mouse HTT. While endogenous mouse HTT levels were independent of age and genotype for R6/2 and BACHD mice, hetQ175 mice showed approximately half the amount of mouse HTT in comparison to respective wild type animals, as the mouse specific assays does not recognize the mutant human/mouse chimeric allele. Background: signal generated by sample buffer. Error bars represent standard deviations of at least three biological replicates. Samples were compared based on a one-way ANOVA statistical analysis: * p < 0.05, **** p < 0.0001, n.s. not significant. (TIF) [file pone.0213521.s008.tif]

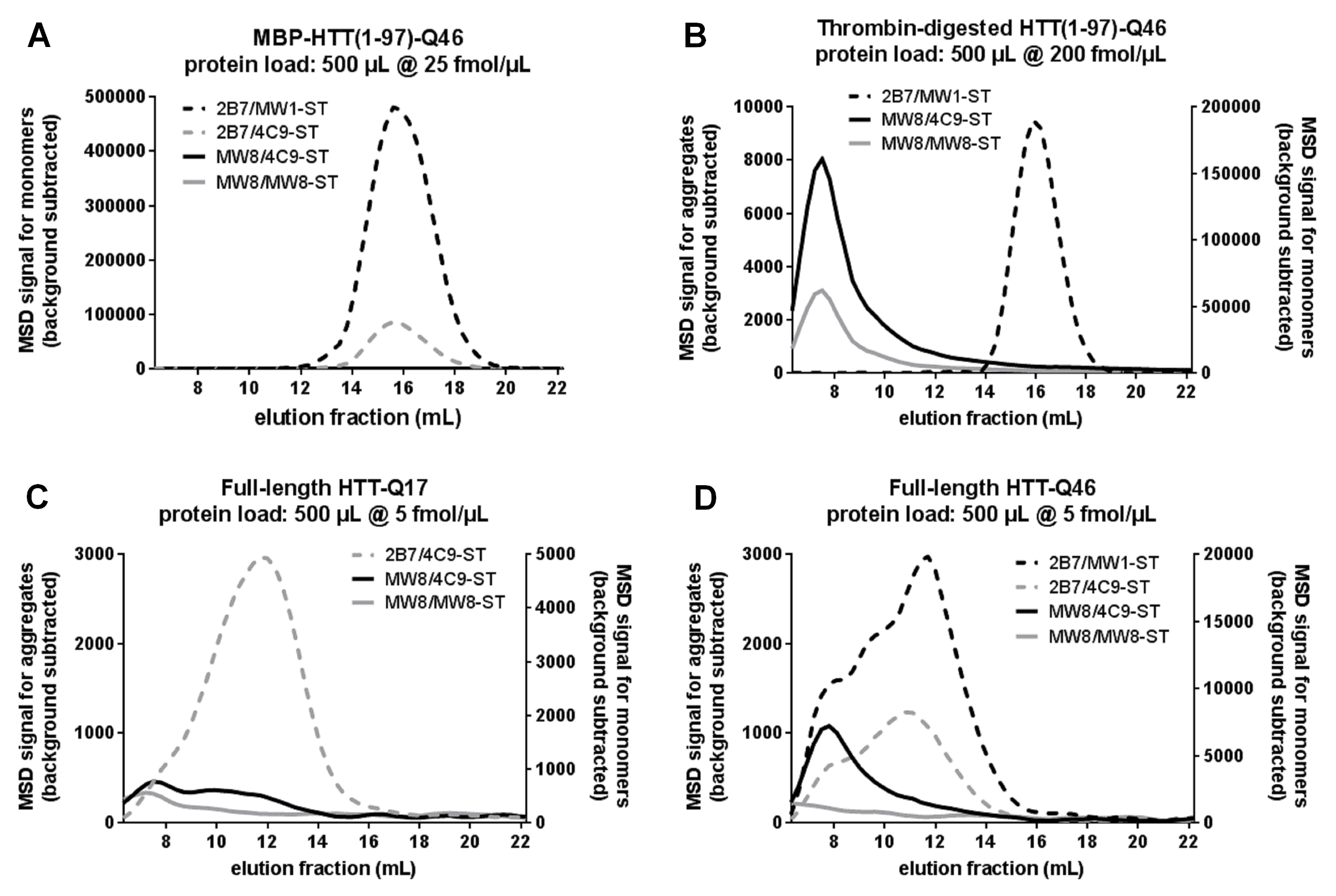

Supplement: S8 Fig — 500 μL of (A) MBP-tagged monomeric HTT(1–97)-Q46, (B) thrombin-digested, aggregated HTT(1–97)-Q46, and full-length HTT (C) HTT-Q17 and (D) Q46 were run on a Superose 6 SEC column at the given concentrations. Here, proteins were separated by size, with larger proteins found in earlier elution fractions. Elution fractions were analyzed in the 2B7 / MW1-ST and/or 2B7 / 4C9-ST assays for human (m)HTT, and the aggregate-selective assays MW8 / 4C9-ST and MW8 / MW8-ST. Aggregated HTT(1–97)-Q46 eluted first at ~ 8 mL, full-length HTT-Q17 and Q46 (Mw: 347 and 351 kDa) showed the maximum elution at ~ 11–12 mL, MBP-HTT(1–97)-Q46 (Mw: 58 kDa) eluted last at ~ 16 mL. Curve shapes were smoothed. Confirmation of detected HTT species by Western blotting of individual elution fractions failed as samples were too dilute after SEC. (TIF) [file pone.0213521.s009.tif]

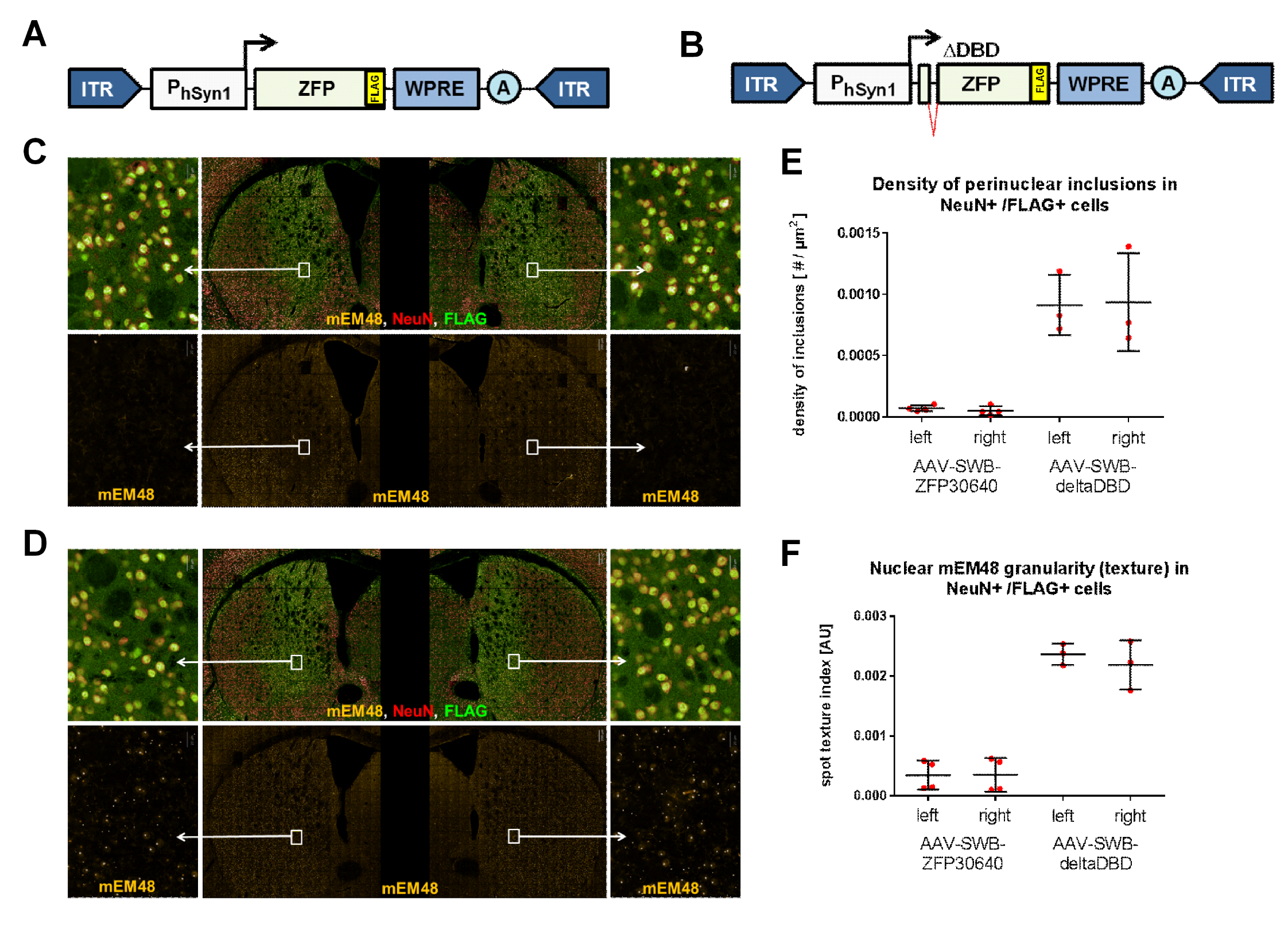

Supplement: S9 Fig — (A) and (B) Schematic representation of ZFP viral vectors: (A) phsyn1-HTT-ZFP30640, (B) ZFP control constructs lacking DNA binding domain, phsyn1-HTT-ZFP-ΔDBD; ITR: inverted terminal repeat, phSyn1: human synapsin1 promoter, ZFP: zinc finger protein, WPRE: woodchuck hepatitis virus posttranscriptional regulatory element, A: polyA sequence, ΔDBD: sequence lacking DNA binding domain. (C and D) Representative images showing mEM48 immunostaining in striatal tissue from heterozygous zQ175 mice injected with (C) AAV-SWB-ZFP30640 and (D) AAV-SWB-ZFP-ΔDBD at 2 months of age and analyzed 4 months post-injection. A strongly reduced HTT inclusions load (mEM48-ir) was observed for AAV-transduced (FLAG+) neurons (NeuN-ir) in ZFP30640-treated animals in comparison with the ΔDBD control. Quantitative analysis of the images shows marked reduction of perinuclear HTT inclusions (E) and nuclear mEM48 granularity (texture) in ZPF30640-treated animals (F). (TIF) [file pone.0213521.s010.tif]
